# *Epilobium* Species: From Optimization of the Extraction Process to Evaluation of Biological Properties

**DOI:** 10.3390/antiox12010091

**Published:** 2022-12-30

**Authors:** Ana-Maria Vlase, Anca Toiu, Ioan Tomuță, Laurian Vlase, Dana Muntean, Tibor Casian, Ionel Fizeșan, George Cosmin Nadăș, Cristiana Ștefania Novac, Mircea Tămaș, Gianina Crișan

**Affiliations:** 1 Department of Pharmaceutical Botany, Faculty of Pharmacy, Iuliu Hațieganu University of Medicine and Pharmacy, 8 Victor Babeș Street, 400012 Cluj-Napoca, Romania; 2 Department of Pharmacognosy, Faculty of Pharmacy, Iuliu Hațieganu University of Medicine and Pharmacy, 8 Victor Babeș Street, 400012 Cluj-Napoca, Romania; 3 Department of Pharmaceutical Technology and Biopharmacy, Faculty of Pharmacy, Iuliu Hațieganu University of Medicine and Pharmacy, 8 Victor Babeș Street, 400012 Cluj-Napoca, Romania; 4 Department of Toxicology, Faculty of Pharmacy, Iuliu Hațieganu University of Medicine and Pharmacy, 8 Victor Babeș Street, 400012 Cluj-Napoca, Romania; 5 Department of Microbiology, Faculty of Veterinary Medicine, University of Agricultural Sciences and Veterinary Medicine, 3-5 Calea Mǎnǎștur, 400372 Cluj-Napoca, Romania

**Keywords:** *Epilobium* species, polyphenols, tocopherols, sterols, oenothein B, LC-MS, biological activity, design of experiments, anticancer activity, ultra-turrax-assisted extraction

## Abstract

*Epilobium* species are used in Romanian folk medicine as tinctures, tea, or tablets for ameliorating the symptoms of benign prostate hyperplasia (BPH), but scientific-based evidence is scarce for this species or other endemic plants of the same genus. Therefore, the aims of this research were to evaluate the phytochemical profile of five endemic *Epilobium* species (*E. hirsutum* L., *E. parviflorum* Schreb., *E. palustre* L. *E. dodonaei* Vill., and *E. angustifolium* L.) and to assess their in vitro biological activity. For enhanced recovery of polyphenols, a D-optimal experimental plan was developed using Modde software and the optimal working conditions were ultra-turrax-assisted extraction, for 8 min, with 30% ethanol in water. The optimized extracts were obtained from various plant parts and were further characterized by LC-MS analysis, with the major compound being oenothein B. All extracts demonstrated good antioxidant activity, evaluated by DPPH and TEAC assays. The most prominent antimicrobial potency of optimized extracts was displayed against *Bacillus cereus*, while against Gram-(+) bacteria, a moderate efficacy was observed. Furthermore, anti-cancer, anti-inflammatory, and antioxidant potential were assessed on normal fibroblasts and prostate carcinoma cell lines. From the evaluated optimized extracts, *E. angustifolium* aerial parts had the highest selectivity toward killing cancerous cells, followed by *E. hirsutum* aerial parts extract. For the antioxidant effect, *E. hirsutum* leaves and *E. hirstum* aerial parts extracts displayed the highest potency, decreasing ROS at the level observed for the positive control. The highest anti-inflammatory potential, based on the IL-6 and IL-8 levels, was displayed by *E. dodonaei* aerial parts and *E. angustifolium* leaves extracts. In conclusion, all five endemic species of *Epilobium* harvested from Romanian flora possess a diverse phytochemical composition, which supports complex biological activities.

## 1. Introduction

The genus *Epilobium* (*Onagraceae*) is distributed worldwide, consisting of over 200 species. Plants belonging to the *Epilobium* genus are erect perennial herbs, often flowering in the first year. Two monophyletic sister genera within *Epilobium-Chamerion* are characterized and the authentication requires a combination of morphological analysis (raphides and trichomes) and TLC [1,2]. Due to frequent interspecific hybridization and similarities between species, taxonomic classification of species belonging to the *Epilobium* genus is considered difficult [2]. Two of the main macroscopic morphological criteria for identification of *Epilobium* species are the height of the stem and the flower symmetry: actinomorphic for species with medium-sized flowers and stems up to 200 cm, as for *E. hirsutum* L., or small flowers with shorter stems, such as *E. parviflorum* Schreb. (stems up to 75 cm) and *E. palustre* L. (stems 5–70 cm), and weakly zygomorphic for species such as *E. angustifolium* L. (stems up to 250 cm) and *E. dodonaei* Vill. (stems 20–110 cm) [3].

*Epilobium* species provide in phytotherapy the product *Epilobii herba*. In 2016, supported by the safe use for at least 30 years, the European Medicines Agency (EMA) released a public document “Herbal medicine: summary for the public” in which it was concluded that the use of willow herb by patients with benign prostatic hyperplasia (BPH) is with the purpose of relieving lower urinary tract symptoms, such as frequent need to urinate or difficulty starting urination [4,5,6,7,8,9]. Willow herb is the common name for the aerial parts of *E. parviflorum* Schreb. and/or *E. angustifolium* L. (syn. *Chamaenerion angustifolium* (L.) Scop.), harvested before or during flowering time [9]. Several *Epilobium* products are commercially available in each EU member state, and they are increasingly applied in the complementary therapy of BPH [7,9,10,11]. 

Up to date, two clinical studies investigated the effect of herbal preparations containing *Epilobium* species for the management of BPH symptoms. Coulson et al. (2013) investigated the effects of a mixture of extracts also containing *E. parviflorum* in a short-term phase II, randomized, double-blind, placebo-controlled clinical trial. The results showed a significant reduction of symptoms in the active group [12]. Esposito et al. (2021) conducted a monocentric, randomized, double-blind, placebo-controlled clinical trial to investigate the effect of *E. angustifolium* on BPH. The study was conducted in 128 adult men who were randomly assigned to the control group (placebo) or test group (*E. angustifolium* extract food supplement) and their findings support the use of this medicinal herb to improve the quality of life for patients as extracts of this species significantly decreased nocturia [7]. 

Moreover, *Epilobium* species demonstrated prominent effects in treating different skin and mucosa diseases and significant anti-diarrheal activity [13,14,15,16,17,18]. 

Plant materials from *Epilobium* sp. are rich sources of secondary metabolites and, lately, a growing interest in the phytochemistry of *Epilobium* sp. has been observed. Bioactive compounds with therapeutic importance were identified in species of this genus, such as phenolic acids and derivatives, flavonoids (flavonol derivatives of quercetin, kaempferol, and myricetin), tannins and related compounds, triterpenes, and steroids, but also other constituents such as fatty acids and amino acids [1,19,20]. As it is found in high concentrations in *Epilobium* sp., the cyclic dimeric ellagitannin oenothein B was concluded to be the major constituent and was claimed to be responsible for many bioactivities of extracts [7,19,21]. Regarding the extraction techniques, mainly maceration (from 1 to 21 days) was used to obtain extracts of *Epilobium* sp. [21,22]. Various solvents were also investigated with relation to extraction yield of bioactive compounds: ethyl acetate, isopropyl alcohol, methanol, methanol/water mixed in various proportions, and water [4,13,16,21].

In Romania, 17 species of *Epilobium* grow in spontaneous flora. Some of them are used in traditional medicine as anti-inflammatory, to treat BPH and associated symptoms, but there are few data on their chemical composition. Up to date, no data were reported for endemic species regarding their content of valuable secondary metabolites such as sterols or the major constituent, oenothein B. This aspect represents a drawback considering that the composition of natural products depends considerably on their geographic origin, even if they are isolated from the same plant species [1,23]. Therefore, one of the aims of this study was the phytochemical characterization of five indigenous *Epilobium* species (*E. hirsutum* L., *E. parviflorum* Schreb.*, E. palustre* L., *E. angustifolium* L., and *E. dodonaei* Vill.), with a focus on polyhenolic content, and secondly on sterolic and tocopherolic compounds. Furthermore, some modern extraction techniques were investigated (ultra-turrax-assisted extraction and ultrasonic-assisted extraction) to find the optimal extraction conditions for extracts rich in polyphenols. Another goal was to assess the differences in the phytochemical composition and biological activities of various plant matrix (roots, where available and sufficient; aerial parts; and leaves, respectively). In addition, the optimized extracts obtained from distinct plant parts were evaluated in various in vitro assays to describe their biological effects.

## 2. Materials and Methods

### 2.1. Chemical and Reagents

The following reagents were used in this study and were purchased from Sigma Aldrich (Sigma Aldrich Chemie GmbH, Schnelldorf, Germany): sodium carbonate, ferric chloride, resazurin, 6-hydroxy-2,5,7,8-tetramethylchromane-2-carboxylic acid (Trolox), 2,2’-azino-bis (3-ethylbenzothiazoline-6-sulfonate (ABTS), dimethyl sulfoxide (DMSO), and phosphate buffer. From Merck (Darmstadt, Germany) were purchased Folin–Ciocâlteu reagent, ethanol, acetonitrile, and methanol. From Carl Roth (Karlsruhe, Germany) was purchased aluminum chloride. All solvents were of LC grade and each of the used reagent was of analytical grade. The water was of Milli-Q-quality.

For spectrophotometric assays and liquid chromatography tandem mass spectrometry (LC-MS/MS) analysis, the following standards were acquired from Sigma Aldrich (Sigma Aldrich Chemie GmbH, Schnelldorf, Germany): apigenin, brassicasterol, caffeic acid, 4-*O*-caffeoylquinic acid, (+)-catechin, caftaric acid, campesterol, chlorogenic acid, *p*-coumaric acid, ergosterol, (−)-epicatechin, fisetin, gentisic acid, hyperoside (quercetin 3-D-galactoside), isoquercitrin (quercetin 3-β-D-glucoside), kaempferitrin, kaempferol, kaempferol-3-rhamnoside, luteolin, myricetin, oenothein B, patuletin, protocatechuic acid, quercetin, quercitrin (quercetin 3-rhamnoside), rutoside (quercetin-3-*O*-rutinoside), stigmasterol, syringic acid, (±)-α-tocopherol, (+)-γ-tocopherol, vanillic acid, vitexin (apigenin 8-C-glucoside), and vitexin 2-O-rhamnoside. From Carl Roth (Karlsruhe, Germany) were purchased beta-sitosterol and sinapic acid, while from Merck (Darmstadt, Germany) were acquired ferulic acid and gallic acid. From Supelco (Bellefonte, PA, USA) was purchased (+)-δ-tocopherol.

For in vitro assays, amoxicillin and miconazole were acquired from Merck KGaA, (Darmstadt, Germany). For cell line testing, Dulbecco’s modified Eagle medium (DMEM) was purchased from Gibco (Paisley, UK), while fetal bovine serum (FBS) and RPMI 1640 (Roswell Park Memorial Institute Medium) were purchased from Sigma Aldrich (Steinheim, Germany).

### 2.2. Plant Material and Preparation for Extraction

Five indigenous *Epilobium* species were harvested from Romanian flora. More precisely, *E. hirsutum* L., *E. parviflorum* Schreb., and *E. palustre* L. were harvested during flowering stage from wild populations near Suceava. *E. angustifolium* L. (formerly known as *Chamaenerion angustifolium* (L.) Scop.) and *E. dodonaei* Vill. (previously known as *Chamaenerion dodonaei* (L.) Scop.) were harvested during flowering stage from higher altitude, from wild populations in Rarău-Giumalău mountains. The exact location and GPS position of the harvesting places are given in Table 1. 

The plant species were authenticated by botany Prof. Gianina Crișan and botany Prof. Mircea Tămaș from the Department of Pharmaceutical Botany, Faculty of Pharmacy, Iuliu Hațieganu University of Medicine and Pharmacy, Cluj-Napoca, and voucher specimens were deposited for each species in the Herbarium of this department (voucher numbers 07/2022 to 11/2022). 

After harvesting, the impurities were removed by washing the plant material with tap water, and then it was conditioned into different organ products, where possible and sufficient (roots, leaves, and aerial parts), and was further air-dried for 5 days, at room temperature (25 °C), safe from sunlight.

### 2.3. Investigation of Optimum Experimental Conditions to Obtain Epilobium sp. Extracts Rich in Phytochemicals

In order to maximize the extraction yield of bioactive compounds, a rational design of experiments was approached. Thus, in the screening step, Modde software v11.0 (Sartorius Stedim Data Analytics AB, Umeå, Sweden) was used to develop a D-optimal experimental design with 3 factors and 3 variables based on which several extracts from the leaves of *E. hirsutum* were obtained (screening step). The following factors with possible influence on the extraction yield were considered: extraction method (qualitative variable), amount of alcohol (ethanol%) in the extraction solvent (quantitative variable), and extraction time (quantitative factor). The responses (output variables) were total polyphenolic content (TPC), total flavonoid content (TFC), and total antioxidant activity (TAA, assessed by the TEAC assay) (Table 2). The *E. hirsutum* leaves extracts had a concentration of 10% (2 g plant material/ 20 mL solvent).

The plant material was ground in a coffee grinder (Bosch TSM6A013B, 75 g, 180 W, Stuttgart, Germany) for 5 min and, next, the powder was sifted through a 200 µm Retsch sieve (Retsch GmbH, Haan, Germany). Vegetal powder (2 g) from *E. hirsutum* leaves was weighed and, next, mixed with the extraction solvent (20 mL) in Falcon tubes. Extraction by ultra-turrax (UTE) was performed by using the Ultra-Turrax homogenizer at 4000 rpm (model T 18, IKA Labortechnik, Staufen, Germany), and ultrasonic-assisted extraction (USE) was carried out with an ultrasonic bath (Sonorex Super RK 100 H, Bandelin Electronic GmbH & Co. KG, Berlin, Germany). The extraction time varied in the range 2–8 min, according to the experimental design. After extraction, the samples were centrifuged for 5 min at 10,000 rpm (SIGMA 3-30KS refrigerated centrifuge, Sigma Laborzentrifugen GmbH, Osterode am Harz, Germany) and the obtained supernatant was recovered and used for spectrophotometric analysis of TPC, TFC, and TAA, and phytochemical analysis by LC-MS/MS. 

After validating the experimental plan, optimum extraction conditions were used to obtain vegetal extracts from the five *Epilobium* sp. from distinct plant parts (roots, leaves, and aerial parts). The optimum extracts were 10% concentration (mass/volume). After centrifugation, the resulting supernatant was recovered and divided in two equal fractions. The first fraction was used to determine the total phenolic content, total flavonoid content, antioxidant activity through TEAC and DPPH assays, phytochemical analysis by LC-MS/MS, and biological activity (antibacterial and antifungal potential). The second fraction was weighed and placed in amber glass vial for lyophilization. The ethanol was evaporated by using a rotary evaporator coupled with vacuum pump (HEI-VAP Advantage Rotary evaporator HL/G1 coupled with Rotavac valve control, Heidolph, Germany). The optimized plant extracts were freezed at −55 °C for 24 h. Then, the samples were lyophilized at pressure 200 mTorr and the temperature was set to −25 °C for another 48 h (SP Scientific Virtis AdVantage 2.0 BenchTop Freeze Dryer/Lyophilizer, Model Advantage Plus EL-85, American Laboratory Trading Inc., East Lyme, CT, USA). The lyophilisates were later used for cell line testing on a normal cell line and a cancerous one. 

### 2.4. Quantitative Determinations of Total Bioactive Compounds and Antioxidant Activity

Total Phenolic Content (TPC)

The following spectrophotometric method was used to determine the total polyphenolic content (TPC): 60 µL of plant extract (diluted 1:50) were mixed in Eppendorf tubes with 270 µL of Folin–Ciocâlteu reagent (diluted with distilled water 1:10) and 270 µL of Na_2_CO_3_ (6% *w/v*) [24]. After incubation at room temperature for 30 min in the dark, the absorbances were recorded at 760 nm (control/solvent blank sample = distilled water) using a Specord^®^ 200 Plus Double-beam Spectrophotometer from Analytik Jena GmbH, Germany. A calibration curve was performed using gallic acid (concentration range for calibration solutions 10–100 µg/mL, R^2^ = 0.9901). The results were expressed as mg gallic acid equivalents (GAE)/mL of plant extract.

Total Flavonoid Content (TFC)

To determine the total flavonoid content (TFC), it was proceeded as previously described: 200 µL plant extract (diluted 1:20) was mixed with 400 µL solution of AlCl_3_ 20 mg/mL in 5% acetic acid in ethanol, prepared according to a ratio of 3:1 (*v/v*) [25]. The absorbance was immediately measured at 420 nm against a solvent blank, with a Specord^®^ 200 Plus Double-beam Spectrophotometer from Analytik Jena GmbH, Germany. The calibration curve was prepared with quercetin and the solutions’ concentrations were in the range 20–150 µM (R^2^ = 0.9894). The results were expressed as mM quercetin equivalents (QAE) per mL of plant extract.

DPPH Radical Scavenging Activity

The antioxidant activity for the optimized extracts of *Epilobium* sp. was evaluated according to a spectrophotometric method previously described [26]. The 2,2-diphenyl-1-picrylhydrazyl (DPPH) assay was employed for this step of the study. The DPPH reagent was dissolved in methanol to reach a concentration of 1 mg/mL. The solution was diluted to 40 pg/mL. Then, 0.8 mL of DPPH solution was mixed with 0.2 mL of plant extract (diluted 1:200) and allowed to incubate in a thermostatic bath at 40 °C for 30 min in the dark. The absorbance change was measured at 517 nm and DPPH reduction capacities were calculated relative to that of the stock solution (40 µg/mL) (A = A_stock solution_ − A_sample_, where A_stock_ solution is the absorbance of DPPH radical + methanol and A_sample_ is the absorbance of DPPH radical + plant extract). The samples’ absorbances were read against a solvent blank by using a Specord^®^ 200 Plus Double-beam Spectrophotometer (Analytik Jena GmbH, Germany). The calibration solutions were prepared in the range 1.22–122 µg/mL, R^2^ = 0.995. Quercetin was used as a reference standard; therefore, the results were expressed as mg quercetin equivalent/mL of plant extract.

Trolox Equivalent Antioxidant Capacity (TEAC) Assay

The determination of total antioxidant activity (TAA) during the screening step of the experimental plan was performed according to the ABTS^•+^ method (2,2’-azino-bis (3-ethylbenzothiazoline-6-sulfonate)) [25]. The spectrophotometric assay is based on the ability of a compound to decolorize the ABTS radical solution (deep green), according to their antioxidant potential and concentration. Reagent 1 (R1) consisted of 0.4 M acetate buffer (pH = 5.8), while reagent 2 (R2) was the 10 mM ABTS^•+^ solution in 30 mM acetate buffer (pH = 3.6). In brief, 12.5 µL of plant extract (diluted 1:200) was mixed with 500 µL R1 and 50 µL R2. The samples were incubated at room temperature for 5 min in the dark before measuring the absorbance at 660 nm against solvent blank (500 µL R1 + 50 µL R2) with a Specord^®^ 200 Plus Double-beam Spectrophotometer from Analytik Jena GmbH, Jena, Germany. The calibration curve was performed with serial dilutions of a water-soluble analogue of vitamin E (Trolox) in the range 0.05–1 mM, dissolved in acetate buffer solution (20 mM, pH = 7.4) (R^2^ = 0.9972). Total antioxidant activity was expressed as mM Trolox equivalent (TE)/L plant extract.

### 2.5. Phytochemical Analysis by LC-MS/MS

Identification and Quantification of Polyphenolic Compounds

The phytochemical profile of the optimized *Epilobium* sp. extracts was assessed by liquid chromatography tandem mass spectrometry (LC-MS/MS) with two distinct analytical methods which were previously validated [27,28]. The following equipment was used: Agilent Technologies 1100 HPLC Series system (Agilent, Santa Clara, CA, USA) equipped with auto sampler, column thermostat, binary gradient pump, degasser, and UV detector. This system was coupled with a mass spectrometer from Agilent, model with Ion Trap 1100 SL (LC/MSD Ion Trap VL, Agilent, Santa Clara, CA, USA). 

The first analytical method which was used was slightly modified (5 new compound standards were added) and was applied to identify 23 polyphenols in optimized *Epilobium* sp. extracts. Briefly, chromatographic separation was performed on a reverse phase analytical column (Zorbax SB-C18, 100 mm × 3.0 mm i.d., 3.5 μm, Agilent Technologies, Santa Clara, CA, USA) with a mobile phase consisting in a mixture of methanol: acetic acid 0.1% (*v/v*) and a binary gradient. Elution began with a linear gradient, initially with 5% methanol and ending with 42% methanol at 35 min. For the next 3 min, isocratic elution followed with 42% methanol. Further, the column was rebalanced with 5% methanol for the following 7 min. The flow rate was set at 1 mL/min, the column temperature was maintained at 48 °C and the injection volume of each sample was 5 µL. Afterwards, the bioactive compounds were detected in both UV and MS mode. Firstly, to detect the polyphenolic acids, the UV detector was set at 330 nm for up to 17 min. Secondly, for detection of the flavonoids and their aglycones, the UV detector was switched to 370 nm wavelength for up to 38 min. The MS system operated using an electrospray ionization source (ESI) in negative mode (capillary +3000 V, nebulizer 60 psi (nitrogen), nitrogen dry gas at 12 L/min, and temperature of 360 °C) [29,30]. 

The second LC-MS validated analytical method was used to identify other 6 polyphenols from optimized *Epilobium* sp. extracts, namely epicatechin, catechin, syringic acid, gallic acid, protocatechuic acid, and vanillic acid, as previously described [31,32]. Chromatographic separation was performed using the same equipment and on the same analytical column mentioned above with the mobile phase a mixture of methanol: acetic acid 0.1% (*v/v*) and a binary gradient as follows: at start—3% methanol; then, at 3 min—8% methanol; next, at 8.5 min—20% methanol, then 20% methanol for up to 10 min, followed by rebalancing of the column with 3% methanol. The flow rate was set at 1 mL/min and the injection volume for each sample was 5 µL. The detection of the bioactive compounds from optimized *Epilobium* sp. extracts was performed in MS mode and the MS system operated using an electrospray ionization source (ESI) in negative mode, according to the technical specifications mentioned in the first analytical method [31,32].

For identification of each bioactive compound from the analyzed plant extracts, the MS spectra/traces were compared with spectra from library. After MS detection, the UV trace was further used for quantification of compounds. For the identified compounds, the calibration curve of their corresponding standards was considered for quantification of their peak areas. 

DataAnalysis (v5.3) and ChemStation (vB01.03) software from Agilent (Santa Clara, CA, USA) were employed for chromatographic data acquisition and interpretation. Results were expressed as micrograms of bioactive compound per gram of dry weight (d.w.) plant sample.

Identification and Quantification of Phytosterols

The phytosterols from optimized *Epilobium* sp. extracts were determined according to a previously validated LC-UV-MS/MS method [33,34,35]. The equipment and the chromatographic analytical column used were the same, but the elution of the compounds was performed in an isocratic manner, with mobile phase acetonitrile: methanol (90:10, *v/v*), a flow rate of 1 mL/min at 45 °C, and 10 μL injection volume. For the detection of compounds in positive ionization, the same mass spectrometer was used, equipped with ion trap and APCI source. The working conditions (nitrogen gas temperature 325 °C, flow rate 7 L/min, nebulizer pressure 60 psi, capillary voltage 4000 V) were carefully adjusted to reach maximum sensitivity. Complete identification of the compounds was performed by comparing retention times and mass spectra with five external standards, namely ergosterol, brassicasterol, stigmasterol, campesterol, and beta-sitosterol. Multiple-reaction monitoring mode (MRM) was used for detection and to avoid or reduce interference [25]. The Agilent ChemStation (vB01.03) software was used for the acquisition of data and the DataAnalysis (v5.3) software was used for the investigation of chromatographic data. The identified phytosterols were further quantified considering the intensity of major daughter ions in the mass spectra. The results were expressed as nanograms phytosterol per gram of dry weight (d.w.) plant sample.

Identification and Quantification of Tocopherols

The tocopherol content (alpha, gamma, delta) for the optimized *Epilobium* sp. extracts was investigated using a previously validated LC-MS/MS analytical method [36]. The same HPLC system was used, coupled with a Brucker Ion Trap SL (Brucker Daltonics GmbH, Leipzig, Germany) and a Zorbax SB-C18 chromatographic column (100 mm × 3.0 mm i.d., 3.5 µm; Agilent Technologies, Santa Clara, CA, USA). Separation was carried out using a mobile phase of water/methanol (7:93; *v/v*) and isocratic elution. The injection volume was 10 µL for the standard solution as well as plant extracts. The thermostat was set at 40 °C and the flow rate was of 1 mL/min. The MS detection was performed in negative ionization, using a source of chemical ionization under atmospheric pressure (APCI) and in multiple-reaction monitoring mode (MRM). The standard solutions of tocopherols (1 mg/mL) were prepared in methanol and the working solutions were obtained by dilution in water / acetone (50:50, *v/v*) [37]. The Agilent ChemStation (vB01.03) and DataAnalysis (v5.3) software were employed for evaluation of chromatographic data. The results were expressed as nanograms tocopherol per gram of dry weight (d.w.) plant sample.

Identification and Quantification of Oenothein B

For the quantification of oenothein B from optimized extracts, a new LC-MS analytical method was developed. An oenothein B standard was used for quantitative determination (manufacturer Sigma-Aldrich, product code 102328214, lot # BCCD7964, CAS 104987-36-2). The equipment used was an Agilent 1100 HPLC Series (with binary pump, autosampler, thermostat, and UV detector), coupled with mass spectrometer (Agilent Ion Trap 1100 SL). A reverse phase analytical column was used (Zorbax SB-C18, 100 mm × 3.0 mm i.d., 3.5 μm, Agilent Technologies, Santa Clara, CA, USA). The mobile phase consisted of a mixture of 0.1% (*v/v*) acetic acid and acetonitrile solution, eluting in gradient after the following program: 92% acetic acid, 0.1% in water (*v/v*), and 8% acetonitrile, then 12% acetonitrile at 2.4 min. The flow rate was 0.8 mL/min, the column temperature was set at 40 °C, the injection volume was 5 µL, and the retention time was 2.4 min. An electrospray ionization source (ESI) was used, with negative ionization mode, nitrogen gas temperature 350 °C, flow rate 12 L/min, nebulizer pressure 60 psi, and capillary potential 5500 V. The analysis mode was SIM (simultaneous ion monitoring); due to the relatively high molecular weight (1596 u.a.m.), the molecule loses two protons, resulting in an ion with two negative charges and m/z 783. The calibration curve was obtained from standard solutions at various concentration levels, representative of the concentration range in the samples. Regression analysis of concentrations for standard solutions (1.3–26 μg/mL) proved good correlation coefficients for the calibration curve. Oenothein B analysis was performed only for optimized extracts and the data was interpreted with Agilent ChemStation (vB01.03) and DataAnalysis (v5.3) software. The results were expressed as milligrams oenothein B per gram of dry weight (d.w.) plant sample.

The validation data (calibration curve, LOD, LOQ, type of MS analysis, retention time, and specific ions for identification) for determination of components in optimized extracts (polyphenols, sterols, and tocopherols) were previously presented [24,26,27,28,29,30,31,32,35].

### 2.6. Principal Component Analysis

Principal component analysis (PCA) was applied to provide an initial overview of the data. Variables describing phytochemical composition and extract properties were scaled to unit variance and principal components were computed to capture a significant amount of input variability. 

For identifying the differences between the investigated observations, hierarchical cluster analysis was performed, having as an input the score vectors of the PCA model. The obtained dendrogram was used to group the observations for the upcoming discriminant analysis, by OPLS-DA modelling (Orthogonal Projections to Latent Structures–Discriminant Analysis). 

The X dataset, represented by extract properties, and the Y dataset, represented by the dummy variable matrix (assigning group membership), were scaled to unit variance. OPLS-DA model performance was evaluated by considering the explained variability for both X and Y (R^2^X, R^2^Y) and through the predictive capacity parameter (Q^2^), determined by full cross-validation. For model interpretation, score and loading plots were generated. All steps were performed with Simca 13 software (Sartorius Stedim Biotech, Göttingen, Germany).

### 2.7. Antimicrobial and Antifungal Activity of the Optimized Epilobium sp. Extracts

Initial in vitro Qualitative Study (Screening)

The antimicrobial potential of the optimized extracts was evaluated in two steps. The initial method was represented by the disk diffusion test, used as a screening method, designed to identify the extracts with increased antimicrobial potential. For this purpose, standard strains of Gram-positive, Gram-negative, and yeasts were selected. Gram-positive bacteria were represented by: *Staphylococcus aureus* ATCC 6538P, *Enterococcus faecalis* ATCC 29212, *Listeria monocytogenes* ATCC 13932, and *Bacillus cereus* ATCC 11778. Gram-negative bacteria used were represented by: *Escherichia coli* ATCC 10536, *Salmonella enteritidis* ATCC 13076, and *Pseudomonas aeruginosa* ATCC 27853, while the yeast strain was *Candida albicans* ATCC 10231. Amoxicillin and Miconazole were used as standard antibacterial and antifungal controls, respectively.

European Committee on Antimicrobial Susceptibility Testing (EUCAST) standard method was used for the screening test, by an adapted disk diffusion technique [38,39]. Microbial colonies, grown on Mueller–Hinton (MH) agar for bacteria and Sabouraud dextrose agar (SDA) for yeast, were suspended in saline to prepare a density of 0.5 on McFarland scale using Densichek device (BioMérieux, Craponne, France). Plastic Petri dishes containing MH agar for bacteria and SDA agar for yeast (8.5 cm diameter) were flooded with the microbial suspension. The excess fluid was removed, and the agar surface allowed to dry. Filter paper discs with 5 mm diameter were placed in a radial model and 20 µL from each extract was placed on the paper disk. The plates were then incubated at 35 ± 2 °C for 18 h for bacteria and at 28 °C for 48 h for the fungal strain [40]. Antimicrobial activity was evaluated as the diameter of the growth inhibition area, measured in mm.

In vitro Quantitative Evaluation

The minimum inhibitory concentration (MIC) method was used for the quantitative assessment and included four Gram-positive standard microbial strains: *Staphylococcus aureus* ATCC 6538P, *Enterococcus faecalis* ATCC 29212, *Listeria monocytogenes* ATCC 13932, and *Bacillus cereus* ATCC 11778. The evaluation was performed according to the adapted EUCAST protocols [40]. Liquid MH medium was used to dilute the extracts in a two-fold serial system, in ten consecutive wells, from the initial (stock = 1/1) to the dilution of 1/512, in 96-wells titer plates. The total broth volume was adjusted to 200 µL/well, with 180 µL from the vegetal extract and 20 µL from the microbial suspension. Positive (broth and microbial inoculum) and negative (30% and 50% ethanol) controls were also used. The plates were incubated at 37 °C for 24 h for bacteria and at 28 °C for 48 h for *Candida albicans*. The MIC values were represented by the lowest concentration of the extract able to inhibit the growth of the microbial cultures (with the same optical density -OD- as the negative control), compared to the positive control, determined by a decreased value of absorbance at 450 nm (HiPo MPP-96, Biosan, Latvia). The MIC_50_ was represented by the MIC value at which ≥50% of the bacterial/yeast cells’ growth was inhibited, determined as the well with the OD value similar to the average of positive and negative control.

### 2.8. In Vitro Biological Activity on Cell Lines

Cell Culture

For the evaluation of the biological activities of the optimized plant extracts, normal human foreskin fibroblasts (BJ) and the LNCaP prostate cancerous cells from ATCC (Manassas, United States of America) were used. The normal cell phenotype was maintained in DMEM (Dulbecco’s modified Eagle’s medium) with low glucose (1 g/L), while the LNCaP cells were cultured in RPMI (Roswell Park Memorial Institute) 1640 with high glucose (5 g/L). Both media were supplemented with 10% FBS (fetal bovine serum). Cells were maintained in a humidified incubator with 5% CO_2_ addition at 37 °C and the media were replaced every other day. At confluency of 70–80%, cells were either subcultured or used in experiments.

Preparation of Extract Solutions

A 100 mg/mL stock solution in dimethyl sulfoxide (DMSO) was obtained by dissolving the lyophilized extracts. The working solutions were prepared by further diluting the stock solution with DMSO. These working solutions were utilized to achieve the necessary concentrations in the cell culture medium, which ranged from 10 to 400 µg/mL.

#### 2.8.1. Anticancer Activity

A number of 30.000 LNCaP cells and 7.500 normal (BJ) cells were seeded in 100 µL in 96-well plates to reach a confluency of 70–80% after 24 h. Dead and unattached cells were removed with phosphate-buffered saline solution (PBS) while the remaining attached cells were exposed for 24 h at a concentration ranging from 10 to 400 µg/mL of extracts. Post-exposure, the cells were washed with PBS and the viability was measured using Alamar Blue (AB) assay, as previously described [31,34,41,42]. AB assay was used to evaluate the metabolic ability of cells to metabolically convert resazurin, a non-fluorescent compound, to resorufin, a fluorescent product. Briefly, cells were incubated for 4 h with a 200 µM resazurin solution, and the fluorescence was measured at λ_excitation_ = 530/25; λ_emission_ = 590/35, using Synergy 2 Multi-Mode Microplate Reader. The experiment was done using three biological replicates, each one including 6 technical replicates. Cells exposed to culture medium containing 0.2% DMSO were used as negative control (NC). The results were calculated as relative values compared to the NC (100%). IC_50_ values were calculated for both cell lines from the dose–effect curves obtained by fitting the data with a 4-parameter logistic curve.

#### 2.8.2. Antioxidant Activities

The ability of the optimized extracts to protect against oxidative stress was evaluated on the normal BJ cell phenotype using the 2,7 dichloro-fluorescein diacetate (DCFH-DA) assay, as presented in previous studies [36,43]. After cellular incorporation, the non-fluorescent DCFH-DA dye is hydrolyzed by intracellular esterases to dichlorodihydrofluorescein (DCFH) which remains confined in the cellular compartment. In the presence of intracellular ROS, DCFH is oxidized to a fluorescent compound that is directly proportional to ROS concentration. Briefly, cells were exposed for 24 h to 3 non-cytotoxic concentrations of the vegetal extracts and further loaded for 2 h with 50 µM DCFH-DA dissolved in Hanks’ balanced salt solution (HBSS). Post-incubation, non-internalized DCFH-DA was washed with PBS 2 and the cells were either exposed to HBSS or 250 µM H_2_O_2_ for 2 h to quantify ROS in non-stimulated and stimulated conditions. The fluorescence was measured using Synergy 2 Multi-Mode Microplate Reader at λ_excitation_ = 485/20 and λ_emission_ = 528/20. The antioxidant potency of vegetal extracts was compared to N-Acetyl Cysteine (NAC) treatment (20 mM solution). Three biological replicates, each with six technical replicates, were used in the experiments. Results were presented as relative values in comparison to the negative control (100%).

#### 2.8.3. Anti-Inflammatory Activities

The anti-inflammatory potentials of the optimized extracts were evaluated by measuring the levels of two pro-inflammatory cytokines, namely IL-6 and IL-8 in the cell culture supernatant using ELISA assays. Normal BJ cells were concomitantly exposed to 100 ng/mL of Lipopolysaccharides (LPS) from *Escherichia coli* and to three non-cytotoxic concentrations of the vegetal extracts (10, 25, and 50 µg/mL) for 24 h, as detailed in former studies [31,43,44]. Following the exposure, cell culture supernatants were aliquoted and stored at –80 °C until the analysis was performed. The concentrations of IL-6 and IL-8 in the cellular supernatants were measured using ELISA kits. A standard curve for each cytokine was included in the experiments, and the cytokine levels were calculated from a 4-parameter logistic curve as recommended in the manufacturer’s instructions.

#### 2.8.4. Statistical Analysis

All data are expressed as mean value ± standard deviations (SD) of three replicates. For the in vitro biological activity on cell lines, experimental data are presented as mean values ± standard deviations (SD) of three biological replicates. Data were statistically analyzed using a One-Way Analysis of Variance (ANOVA) with a Holm–Sidak post hoc test. Graphical representation and data analysis were performed using the SigmaPlot 11.0 software (Systat, Software Inc., Chicago, IL, USA). Results were considered statistically different if *p* values were lower than 0.05.

## 3. Results and Discussions

### 3.1. Fitting the Experimental Data with the Models

The matrix of the experimental design which consisted of 19 vegetal extracts of *E. hirsutum* leaves was generated by the Modde software. In Table 3 are summarized the outcomes after performing the experimental runs.

### 3.2. The Influence of Experimental Conditions on TPC, TFC, and TAA

The experimental design was fitted with the obtained data by the partial least squares regression (PLS) method in the screening step. The fitting parameters for the output variables (TPC, TFC, and TAA) are shown in Table 4. For all evaluated variables, a good fit was obtained (R^2^ between 0.848 and 0.962); in addition, adequate model validity and good reproducibility were also observed. The statistical parameter that overestimates the goodness of fit is R^2^; more precisely, it describes the percent of response’s variation which is explained by the chosen model, and for a good model it should be close to a value of 1. On the other hand, Q^2^ indicates the percent of response’s variation that is predicted by the model according to cross-validation. High values for these two statistical parameters and a difference of 0.2–0.3 between them indicates a high predictive power for an adequate model. For the chosen model, even though the difference between these two statistical parameters was higher than 0.3, a good reproducibility was obtained, and a good model validity. For TAA, the model validity was lower because it was noticed in spectrophotometric assays that for the *E. hirsutum* leaves extracts, both polyphenols and flavonoids display good antioxidant activity; therefore, it is a synergistic mechanism of action. On the other hand, it was observed that polyphenols and flavonoids exhibit high extraction yields under different working conditions, and for the optimization step, it was decided to determine the optimum extraction conditions which assure a maximum recovery yield for polyphenols, as it was proved by other authors that these are the most prominent bioactive compounds responsible for the biological activity of *Epilobium* sp. [45]. However, for comparison reasons, an *E. hirsutum* leaves extract with EtOH 50% was also obtained and evaluated in the following biological assays, considering that, in this case, a good recovery was noticed for both polyphenols and flavonoids, and an adequate total antioxidant activity. This extract was obtained under the optimum extraction method as given by the Modde software after maximizing the TPC and TAA, namely ultra-turrax-assisted extraction (UTE) for 8 min.

**Figure 1:**
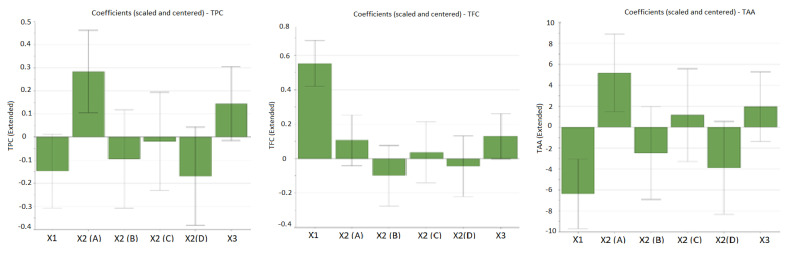
The influence of working conditions on TPC (total phenolic content), TFC (total flavonoid content), and TAA (total antioxidant activity) by TEAC assay upon *E. hirsutum* leaves extracts during the screening step, depicted as scaled and centered coefficient plots. X_1_-ethanol ratio (%, *v/v*); X_2_-extraction methods: A-ultra-turrax extraction (UTE); B-ultrasonic-assisted extraction (USE); C-maceration (48 h) followed by UTE; D-maceration (48 h) followed by USE. X_3_-extraction time (min).

As can be observed from Table 3, the working conditions influence the extraction yield of bioactive compounds. The influence of working conditions on TPC, TFC, and TAA are shown in the form of diagrams with scaled and centered coefficients in Figure 1.

From the figures given above, it can be noticed that the factor with the highest influence on the recovery of bioactive compounds is the percentage of alcohol (ethanol) in the extraction solvent (input variable X_1_). In the case of polyphenols, a good extraction yield was obtained when a hydroalcoholic mixture with 30% ethanol (EtOH) was used, and the extraction yield decreased as the percentage of ethanol in the solvent mixture increased (negative influence). Significant antioxidant activity was obtained in the case of extracts obtained in this solvent mixture (EtOH 30%), confirming that polyphenols are the main bioactive compounds that provide the antioxidant potential of *E. hisutum* leaves extracts. In the case of flavonoids, the extraction was favored in a hydroalcoholic mixture with 70% EtOH, thus having a positive influence on the extraction yield. However, no increased TAA was obtained for flavonoid-rich extracts; thus, for the optimization step, the maximization of TPC and TAA was chosen. 

The extraction method displayed a positive influence upon the extraction yield; high values for TPC and TAA were observed when using UTE (samples N1, N9, N10, N17, and N19). The extraction time exhibited a positive influence upon all evaluated output variables, but it was more marked for TPC (samples N9 and N17). 

### 3.3. Investigation of Optimum Experimental Conditions to Obtain Epilobium sp. Extracts Rich in Phytochemicals

In the optimization step, the goal was to maximize the extraction of polyphenols (TPC), which ensure a marked antioxidant activity (evaluated as TAA). Thus, the Modde software returned the optimal working parameters as following: ultra-turrax-assisted extraction (4000 rpm), for 8 min, in hydroalcoholic mixture of solvents with 30% ethanol. Under these conditions, the main bioactive compounds were extracted from the other harvested species, from various parts of the plants. In this stage, nine different extracts of 10% plant product concentration (mass/volume) were obtained, as follows: *E. hirsutum* leaves (50% EtOH—this extract was made in order to have one sample with higher flavonoid content in the series of optimal extracts, for the in vitro biological testing), *E. hirsutum* leaves (EtOH 30%), *E. hirsutum* aerial parts (EtOH 30%), *E. hirsutum* roots (EtOH 30%), *E. parviflorum* aerial parts (EtOH 30%), *E. palustre* aerial parts (EtOH 30%), *E. dodonaei* aerial parts (EtOH 30%), *E. angustifolium* leaves (EtOH 30%), and *E. angustifolium* aerial parts (EtOH 30%).

For the nine optimized *Epilobium* sp. extracts, the following determinations were performed: total polyphenol content (TPC), total flavonoid content (TFC), antioxidant activity by TEAC and DPPH assays, detailed phytochemical profile with identification, and quantification of polyphenols, tocopherols, and sterols (using previously validated LC-MS/MS analytical methods). Biological properties were also evaluated for the optimized extracts: antimicrobial activity on Gram-positive and Gram-negative bacteria, and antifungal potential. In addition, cytotoxic, antioxidant, and anti-inflammatory activity were determined in vitro in studies performed on a normal human fibroblast cell line and a human prostate carcinoma cell line.

### 3.4. Quantitative Determinations of Total Bioactive Compounds and Antioxidant Activity

Table 5 summarizes the results obtained for the spectrophotometric assays performed on the optimized vegetal extracts. The experiments were performed in triplicate (n = 3) and the results are given as mean value ± standard deviation (SD).

### 3.5. Phytochemical Analysis by LC-MS/MS

Table 6 presents the quantitative and qualitative determination for individual polyphenols, tocopherols, sterols, and the main component found in *Epilobium* species, oenothein B, respectively. The corresponding UV chromatograms for individual polyphenolic compounds quantified by the first LC-MS method, the total ion chromatogram (TIC) for polyphenols analyzed with the second analytical method, and the TIC for tocopherols are provided in Supplementary material (Figures S1–S9).

To the best of our knowledge, this is the first study to present the detailed phytochemical profile for *E. palustre* and *E. dodonaei*, and the first research to investigate the content of sterols and tocopherols in five distinct *Epilobium* sp. Other scientific articles published recently which mentioned these two categories of bioactive compounds did not specify the exact amount determined in various plant parts of *Epilobium* sp. For tocopherol analysis, a study mentioned the α-, γ-, and δ-tocopherol content determined in seed oil of *E. dodonaei*, *E. hirsutum*, and *E. parviflorum*, but currently the seed oil of these species is not used for medicinal purpose, the aim of that study being the general screening of the phytocomplex [46].

Concerning the polyphenols analysis, the results given in Table 6 support previous findings, in which the main bioactive compounds of this category were hyperoside, myricetin, quercitrin, and kaempferol-3-rhamnoside [47]. In addition to what was presented to date in the scientific literature, the present study determined in all five *Epilobium* sp. harvested from Romanian flora and in all investigated plant parts (leaves, aerial parts, roots) high concentrations of caftaric acid (1002.29 ± 57.94 μg/g d.w. for *E. parviflorum* aerial parts EtOh 30%—highest amount determined) and gallic acid (662.28 ± 42.46 μg/g d.w. in *E. dodonaei* aerial parts EtOH 30%—highest concentration observed). Furthermore, the content of sterols was investigated, with beta-sitosterol found in highest concentrations (ranging from 1206.29 ± 80.12 ng/g d.w. for *E. hirsutum* leaves EtOH 30% to 2694.80 ± 152.71 ng/g d.w. for *E. angustifolium* aerial parts EtOH 30%). The analytical standards for tocopherol identification were α-, γ-, and δ-tocopherol, of which α- and δ-tocopherol were quantified in all extracts. *E. parviflorum* aerial parts EtOH 30% contained the highest amount of alfa-tocopherol (9435.45 ± 398.51 ng/g d.w.) and delta-tocopherol (572.76 ± 33.14 ng/g d.w.).

From the investigated extracts, *E. hirsutum* roots EtOH 30% displayed a modest phytocomplex, poor in bioactive compounds which, where present, were determined in low concentrations. It was also noticed that the main bioactive compound, oenothein B, was absent from this species plant part. Oenothein B is supposed to be involved in anti-inflammatory activity of *Epilobium* sp. extracts, due to its urolithins metabolites formed by gut microbiota after oral administration of herbal supplements [4]. On the other hand, Lin et al. (2022) assessed the anti-inflammatory activity of an ethanol fraction of *E. angustifolium* from which oenothein B was removed and the activity was much stronger than that of oenothein B [48]. This suggests a synergistic mechanism of action of the plant extract, which should possess a phytocomplex rather than a higher concentration in a certain bioactive compound. For these reasons, in further in vitro studies performed on cell lines, the *E. hirsutum* roots EtOH 30% was not considered for evaluation. The highest amount of this ellagitannin was obtained in *E. dodonaei* aerial parts EtOH 30% (106.82 ± 7.45 mg/g d.w.), *E. parviflorum* aerial parts EtOH 30%) (98.84 ± 5.89 mg/g d.w.), and *E. hirsutum* aerial parts EtOH 30% (73.49 ± 3.89 mg/g d.w.).

The influence of the extraction solvent mixture was observed when comparing the phytocomplex of *E. hirsutum* leaves EtOH 50% extract and *E. hirsutum* leaves EtOH 30%. 

Although higher concentrations were obtained for certain polyphenols when EtOH 50% was used (*p*-coumaric acid, hyperoside, myricetin, quercitrin, quercetol, kaempferol-3-rhamnoside, epicatechin, catechin, and oenothein B), some bioactive compounds were absent and were quantified only in *E. hirsutum* leaves EtOH 30% extract (gentisic acid, beta-sitosterol, campesterol, and γ-tocopherol).

### 3.6. Principal Component Analysis 

**Figure 2:**
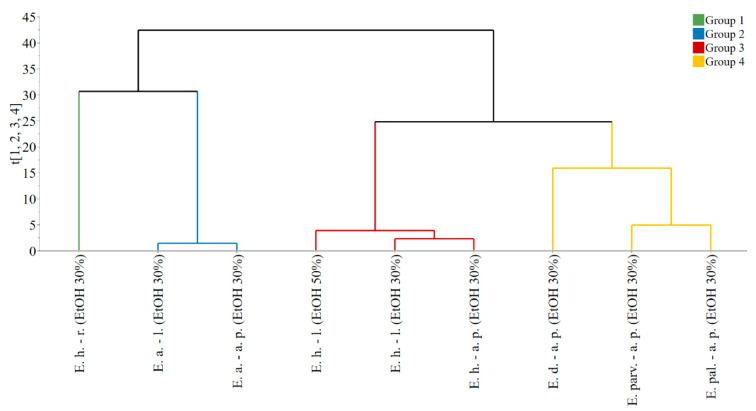
Dendrogram presenting the grouping of observations based on the score vectors of the PCA model. Legend: E. h.–l.—*E. hirsutum* leaves. E. h.–a. p.—*E. hirsutum* aerial parts. E. h.–r.—*E. hirsutum* roots. E. parv.–a. p.—*E. parviflorum* aerial parts. E. pal.–a. p.—*E. palustre* aerial parts. E. d.–a. p.—*E. dodonaei* aerial parts. E. a.–l.—*E. angustifolium* leaves. E. a.–a. p.—*E. angustifolium* aerial parts. EtOH 50%—ethanol in water 50/50 (*v/v*). EtOH 30%—ethanol in water 30/70 (*v/v*).

The PCA model, fitted on the dataset comprised of extract composition and properties, presented four principal components, explaining a total of 91.8% data variability. The obtained score vectors allowed the identification of four groups, as it can be observed from the dendrogram of the hierarchical clustering analysis (Figure 2).

To compare the investigated observations, an OPLS-DA model was fitted to identify differences in extract composition and properties. The fitted model presented excellent performance, reflected by the large amount of variability from the X matrix that contributed to class discrimination (R^2^X predictive—73.8%), combined with the high values of R^2^Y (0.996) and Q^2^ (0.902). The R^2^Y parameter reveals the explained variability in the Y matrix, which contains class membership, whereas Q^2^ reveals the predictive power of the model. Both parameters suggest that a good discrimination was achieved [49,50].

**Figure 3:**
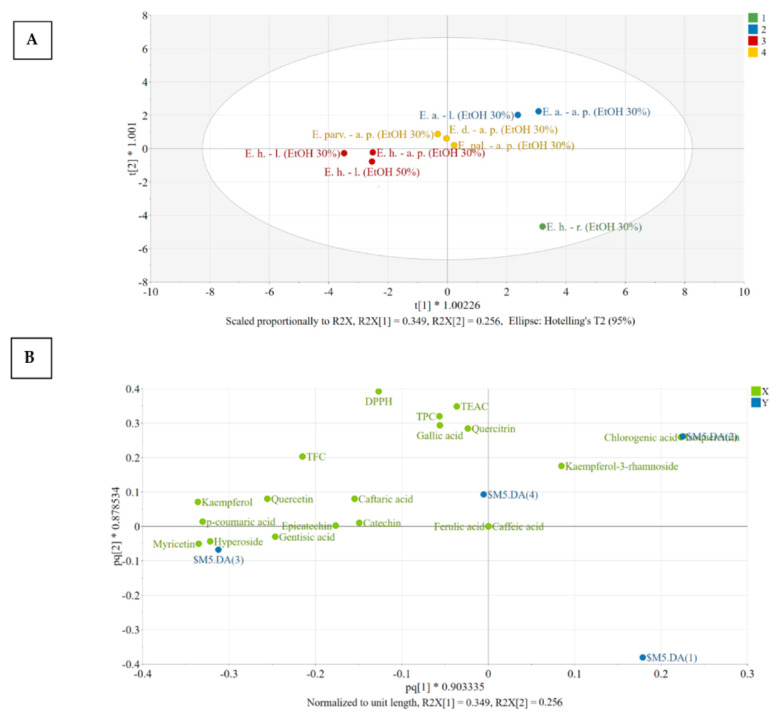
The score (**A**) and loading (**B**) scatter plots of the OPLS-DA model. Legend: E. h.–l.—*E. hirsutum* leaves; E. h.–a. p.—*E. hirsutum* aerial parts; E. h.–r.—*E. hirsutum* roots; E. parv.–a. p.—*E. parviflorum* aerial parts; E. pal.–a. p.—*E. palustre* aerial parts; E. d.–a. p.—*E. dodonaei* aerial parts; E. a.–l.—*E. angustifolium* leaves; E. a.–a. p.—*E. angustifolium* aerial parts; EtOH 50%—ethanol in water 50/50 (*v/v*); EtOH 30%—ethanol in water 30/70 (*v/v*).

For interpretation purposes, the score and loading plots of the OPLS-DA model were generated (Figure 3). Figure 3A reveals the distribution of observations in the plane of the first two predictive score vectors of the model, whereas Figure 3B explains the distribution found in the score plot. Extracts presenting a similar placement in the score plot have similar characteristics, whereas an increasing distance between points suggests increasing differences. 

To understand the contribution of variables in class discrimination, the positioning of the X variables (green) in relation to each other and to the dummy variables (blue) was evaluated. A variable has a good discriminating power if it is positioned far from the origin and close to a certain dummy variable [49,50]. Clustering of different variables reveals a strong positive correlation. In this respect, chlorogenic acid and isoquercitrin correlated and are characteristic of the members of Class nr 2, both aerial parts and leaves of *E. angustifolium* being characterized by large amounts of these constituents. Ferulic acid was absent from these extracts and caffeic acid could only be identified based on MS spectra, but not quantified (<LOQ), as they appear close to the origin of the loading plot. 

Four distinct groups could be identified after PCA (Figure 3A), namely:

Group 1: [*E. hirsutum* roots EtOH 30%]: the extract *E. hirsutum* roots has very low number of bioactive compounds and thus presents low activity, as can be seen from the positioning of the dummy variable ($M12.DA(1)). The dummy variable of group number 1 is found in the lower right quadrant of the loading scatter plot, having no other variables displaced in this region.

Group 2: [*E. angustifolium* aerial parts EtOH 30%; *E. angustifolium* leaves EtOH 30%]: the members of group 2 have large amounts of chlorogenic acid and isoquercitrin, above average amount of quercitrin, and antioxidant activity evaluated by TEAC and DPPH assays. In addition, this group presents lower than average amounts of the variables found in the opposite side of the plot, in the direction of the horizontal axis, namely: caftaric acid, gentisic acid, *p*-coumaric acid, hyperoside, and kaempferol. 

Group 3: [*E. hirsutum* leaves EtOH 50%/EtOH 30%; *E. hirsutum* aerial parts EtOH 30%]: the members of group 3 have larger amounts of the variables found in the close region of $M12.DA(3) dummy variable, such as hyperoside, *p*-coumaric acid, kaempferol, quercetol, gentisic acid, and lower amount of kaempferol-3-rhamnoside. 

Group 4: [*E. dodonaei* aerial parts EtOH 30%; *E. parviflorum* aerial parts EtOH 30%; *E. palustre* aerial parts EtOH 30%]: the members of group 4 have an above-average content of caftaric acid with variable content of quercitrin (*E. dodonaei > E. parviflorum > E. palustre*) and kaempferol-3-rhamnoside (*E. dodonaei > E. parviflorum > E. palustre*). 

### 3.7. Antimicrobial and Antifungal Activity of the Optimized Epilobium sp. Extracts

Initial in vitro Qualitative Study (Screening)

Antimicrobial activity was evaluated using four Gram-positive bacterial strains, three Gram-negative, and one yeast strain. This initial step was designed to select the extracts with increased antimicrobial potential and the susceptibility of groups of micro-organisms. The antimicrobial activity against Gram-negative bacteria was absent and the evaluation further focused on Gram-positive and yeasts. An increased efficiency was observed against Gram-positive bacteria and yeasts, as observed in Table 7. The potential against Gram-positive bacteria was moderate, with inhibition areas ranging from 8 to 15 mm, while for *Candida albicans,* it was between 9.82 and 13.66 mm. The activity was increased against *Bacillus cereus* strain, compared to the other Gram-positive strains and antibiotic positive control (amoxicillin).

In vitro Quantitative Evaluation

The initial screening demonstrated moderate antimicrobial potential against Gram-positive bacteria, and the quantitative antimicrobial potential using MIC method was tested in this second step. The MIC values ranged from 1/32 to 1/256, as observed in Table 8, with good overall efficiency against *Bacillus cereus* and *Enterococcus faecalis*.

The presented results confirm the antimicrobial potential of *Epilobium* species which was formerly investigated on various bacterial, fungal, and viral strains [1,19,51]. Previous studies mainly focused on the antibacterial potential of *E. angustifolium* extracts which proved to be efficient against Gram+ and Gram- bacteria; recent studies also confirmed the antimicrobial potential of *E. parviflorum* and *E. hirsutum* [1,16,52,53,54]. *E. angustifolium* and *E. parviflorum* extracts inhibited the growth of some fungi, including *Candida albicans*. In addition to what was researched to date, the antimicrobial effect of *E. palustre* and *E. dodonaei* was evaluated, and for all five endemic *Epilobium* species, this biological effect was assessed on ethanolic extracts (30%). For *E. hirsutum*, the extracts of leaves with 30% and 50% ethanol in water were tested; the results were not significantly different except for the activity upon *Enterococcus fecalis*, where *E. hirsutum* leaves (EtOH 50%) displayed a higher inhibition diameter versus the EtOH 30% extract (9.65 mm vs. 6.62 mm).

### 3.8. In Vitro Biological Activity on Cell Lines

#### 3.8.1. Anticancer Activity 

**Figure 4:**
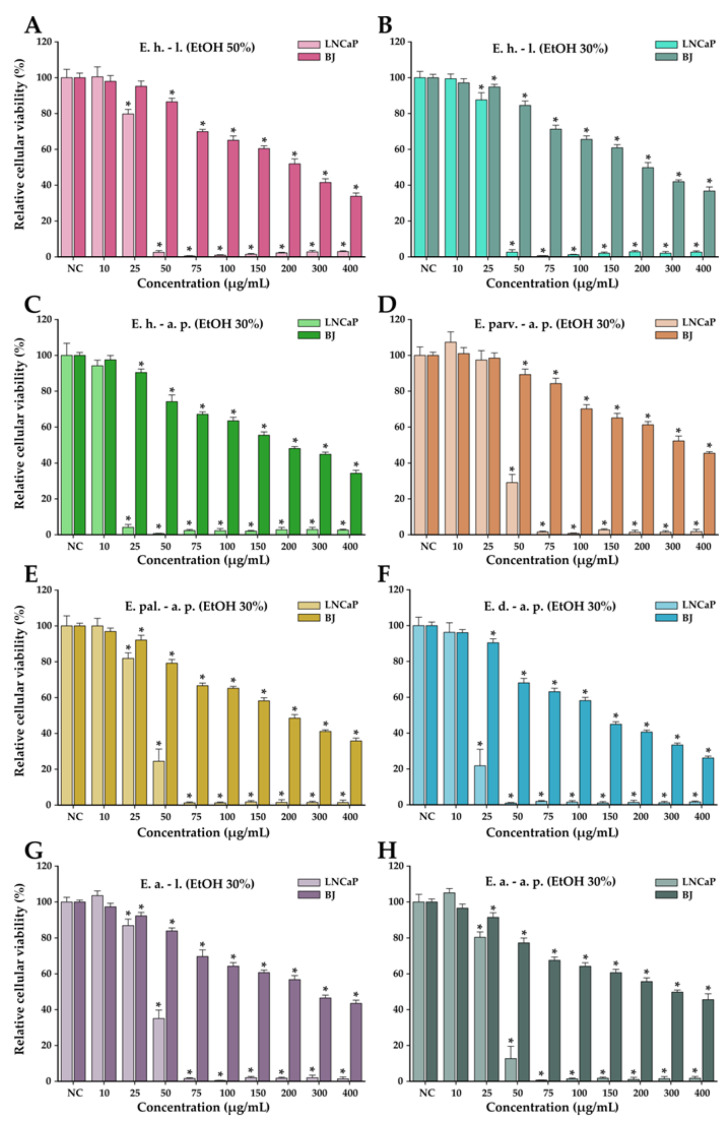
Cytotoxicity of *E. hirsutum* leaves EtOH 50% (**A**—E. h.–l.), *E. hirsutum* leaves EtOH 30% (**B**—E. h.–l.), *E. hirsutum* aerial parts EtOH 30% (**C**—E. h.–a.p.), *E. parviflorum* aerial parts EtOH 30% (**D**—E. parv.–a. p.), *E. palustre* aerial parts EtOH 30% (**E**—E. pal.–a. p.), *E. dodonaei* aerial parts EtOH 30% (**F**—E. d.–a. p.), *E. angustifolium* leaves EtOH 30% (**G**—E. a.–l.), and *E. angustifolium* aerial parts EtOH 30% (**H**—E. a.–a. p.) evaluated using Alamar Blue assay on the LNCaP prostate carcinoma cell line and normal BJ human foreskin fibroblasts after a 24 h exposure. The results are expressed as the means ± standard deviations of three biological replicates (each one including 6 technical replicates). The values were calculated as relative values compared to the negative control (NC) (100%). Asterisks (*) indicate statistically significant differences in comparison with negative control (ANOVA + post hoc Holm–Sidak test at *p < 0.05*).

**Figure 5:**
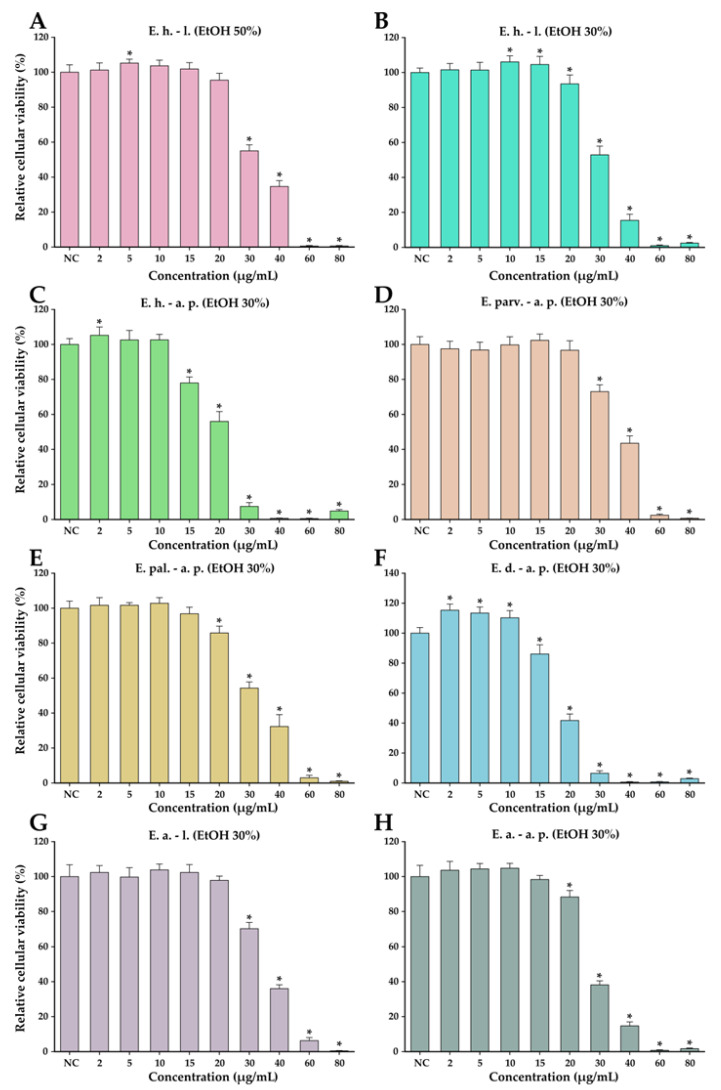
Cytotoxicity of *E. hirsutum* leaves EtOH 50% (**A**—E. h.–l.), *E. hirsutum* leaves EtOH 30% (**B**—E. h.–l.), *E. hirsutum* aerial parts EtOH 30% (**C**—E. h.–a.p.), *E. parviflorum* aerial parts EtOH 30% (**D**—E. parv.–a. p.), *E. palustre* aerial parts EtOH 30% (**E**—E. pal.–a. p.), *E. dodonaei* aerial parts EtOH 30% (**F**—E. d.–a. p.), *E. angustifolium* leaves EtOH 30% (**G**—E. a.–l.), and *E. angustifolium* aerial parts EtOH 30% (**H**—E. a.–a. p.) evaluated using Alamar Blue assay on the LNCaP prostate carcinoma cell line after a 24 h exposure (after diluting the working solutions 1:5 in DMSO). The results are expressed as the means ± standard deviations of three biological replicates (each one including 6 technical replicates). The values were calculated as relative values compared to the negative control (100%). Asterisks (*) indicate statistically significant differences in comparison with negative control (ANOVA + post hoc Holm–Sidak test at *p < 0.05*).

The anticancer potential of the *Epilobium* species extracts was evaluated on the LNCaP cancerous cells in parallel with normal human foreskin fibroblasts (BJ). This potential was firstly evaluated in a concentration range from 10 to 400 µg/mL in both cell types (Figure 4). In general, a cytotoxic effect was observed starting from the dose of 25 µg/mL in both cell types; however, at higher doses, a large discrepancy was observed between the recorded viabilities of the cancerous and normal cell phenotypes. At the dose of 50 µg/mL, the recorded viabilities on the LNCaP cells varied from approximately 0 to 35%, while in the case of normal cells, the viability varied from 90 to 100% (Figure 4). At the highest concentration tested of 400 µg/mL, the viability in the case of normal cells was between 25 and 45%, depending on the extract tested, with *E. dodonaei* aerial parts (EtOH 30%) extract displaying the highest cytotoxicity. From the extracts tested, *E. dodonaei* aerial parts (EtOH 30%) extract appeared to bear the highest cytotoxicity towards the cancerous cell phenotype, decreasing the cellular viability to approximately 20% at a dose of 25 µg/mL (Figure 4 and Figure 5). 

For an easier comparison of biological potencies, IC_50_ values were calculated for all extracts on both cell types (Table 8). For the BJ cell line, the IC_50_ was calculated based on the data obtained for the 10–400 µg/mL concentration range, while in the case of the LNCaP cells, the concentration range was adjusted to 2 to 80 µg/mL by further diluting the working solutions 1:5 in DMSO (Figure 5). The values obtained indicate higher cytotoxicity for the cancerous cell line, the IC_50_ for the cancerous cell line being 5 to 10 times lower than for their normal counterparts. In addition, the higher cytotoxic potency observed for *E. dodonaei* aerial parts (EtOH 30%) extract was demonstrated, this extract having the IC_50_ values of 18.38 µg/mL and 126.05 µg/mL for LNCaP and BJ cells, respectively (Table 8). To evaluate if there is a statistical difference between the cytotoxic potencies of the extracts, an ANOVA 2-Way analysis was performed for each cell line, using as variables the type of the extract and the concentration tested and, as the response, the measured viability. Based on this analysis, the potency of the extracts was statistically different for almost all cases (no statistical difference for LNCaP cell line between *E. parviflorum* aerial parts EtOH 30% vs. *E. angustifolium* aerial parts EtOH 30% and *E. hirsutum* leaves EtOH 30% vs *E. palustre* aerial parts EtOH 30%; no statistically significant difference for BJ cell line between *E. hirsutum* leaves EtOH 30% vs *E. hirsutum* leaves EtOH 50%). 

A selectively cytotoxic effect against cancerous cell phenotypes is mandatory for substances with possible antitumoral activity. In this regard, the ratios between the extrapolated IC_50_ for cancerous and normal cells were calculated (Table 9). Of the investigated extracts, *E. angustifolium* aerial parts EtOH 30% had the highest selectivity toward killing cancerous cells, followed by *E. hirsutum* aerial parts EtOH 30% extract. In the case of *E. angustifolium* aerial parts EtOH 30%, the IC_50_ obtained for normal cells was more than 10 times higher than the IC_50_ obtained for LNCaP cells. Even though *E. dodonaei* aerial parts EtOH 30% extract had the highest cytotoxicity toward both cell lines, the selectivity towards the cancerous cell phenotype was low.

The present findings are consistent with previous studies on this species, as *Epilobium* preparations are traditionally recognized as a remedy for prostate impairment and numerous studies refer to this application [7,55]. The in vivo investigation of *E. parviflorum* extracts on 5-α-reductase and aromatase activity showed that only the aqueous extract was active, and the following research led to the identification of oenothein B as active principle [56,57]. Nonetheless, in this study, a good antiproliferative effect was obtained for the ethanolic (30%) extract of *E. parviflorum* (Figure 4). Vitalone et al. (2003) observed a specific and significant anti-proliferative effect of *E. angustifolium*, *E. hirsutum*, and *E. tetragonum* ethanolic extracts. However, the selectivity of the anti-proliferative effect on other tumor cells lines was investigated and its activity proved not to be prostate-specific [58]. Stolarczyk et al. (2013) noticed a dose dependent inhibition of PSA secretion and arginase activity of aqueous extracts from *E. angustifolium*, *E. parviflorum*, and *E. hirsutum* [4]. On the other hand, the lack of information regarding oenothein B bioavailability makes it difficult to estimate its real role in the activity of extracts. 

#### 3.8.2. Antioxidant Activities

**Figure 6:**
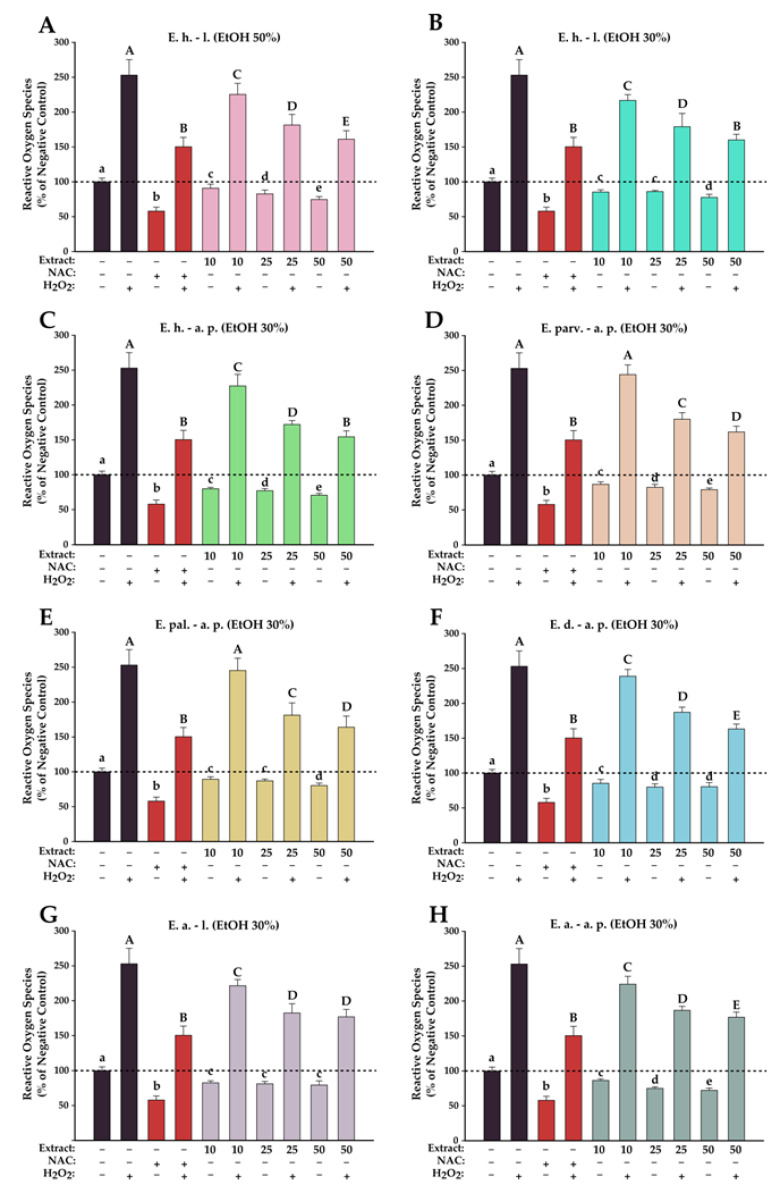
Antioxidant effects of *E. hirsutum* leaves EtOH 50% (**A**—E. h.–l.), *E. hirsutum* leaves EtOH 30% (**B**—E. h.–l.), *E. hirsutum* aerial parts EtOH 30% (**C**—E. h.–a.p.), *E. parviflorum* aerial parts EtOH 30% (**D**—E. parv.–a. p.), *E. palustre* aerial parts EtOH 30% (**E**—E. pal.–a. p.), *E. dodonaei* aerial parts EtOH 30% (**F**—E. d.–a. p.), *E. angustifolium* leaves EtOH 30% (**G**—E. a.–l.), and *E. angustifolium* aerial parts EtOH 30% (**H**—E. a.–a. p.) evaluated using DCFH-DA assay on normal BJ human foreskin fibroblasts cell line. Cells were incubated with the extracts (10, 25, and 50 µg/mL) or NAC (20 mM) for 24 h and further exposed to 50 µM DCFH-DA. The antioxidant potential was measured in stimulated/non-stimulated conditions after a 2 h exposure in the presence and absence of 250 µM H_2_O_2_. Data are presented as relative means ± standard deviation of three biological replicates. All values are expressed as relative values compared to the negative control (100%). Different letters (a–e in non-stimulated conditions, and A–E in stimulated conditions) show statistically significant differences (ANOVA + Holm–Sidak post hoc test at *p < 0.05*).

The antioxidant effects of the *Epilobium* sp. extracts were evaluated on the normal fibroblast cells at three concentrations (10, 25, and 50 µg/mL) that did not induce significant toxicities after an exposure of 24 h. The antioxidant activities were evaluated in two conditions, namely H_2_O_2_-stimulated and non-stimulated conditions. In non-stimulated conditions, exposure to the extracts induced a statistical decrease in ROS that was in almost all cases dose-dependent (Figure 6). In comparison with the positive control (NAC), the antioxidant properties of the extracts were more modest; no extract, independent of the dose tested, displayed a capacity to neutralize ROS such as NAC. In stimulated conditions, exposure of BJ to H_2_O_2_ resulted in an approximately 2.5-times increase in the quantity of ROS, while pre-incubation with the antioxidant NAC partially mitigated this increase (1.5-times increase). Similar to the results obtained in non-stimulated conditions, the *Epilobium* sp. extracts presented an antioxidant potential, and exposure to the extracts resulted in a dose-dependent decrease in ROS. From the extracts evaluated, *E. hirsutum* leaves EtOH 30% and *E. hirsutum* aerial parts EtOH 30% displayed the highest potency, decreasing ROS at 50 µg/mL in stimulated conditions at the level observed for the positive control.

To date, no data could be found in the scientific literature regarding the antioxidant potential of *E. palustre* and *E. dodonaei*, the current study being the first to report this biological activity for these species. Regarding *E. hirsutum*, *E. parviflorum*, and *E. angustifolium* extracts activity, this study was the first to evaluate the antioxidant potential using the 2,7 dichloro-fluorescein diacetate (DCFH-DA) assay on the normal BJ cell phenotype. Merighi et al. (2021) reported that *E. parviflorum* extract at 1 µg/µL and 0.1 µg/µL concentrations significantly decreased the H_2_DCF-DA absorbance increased by LPS in RAW 264.7 macrophage murine cells, supporting the antioxidant property of this species [22]. Previous studies confirmed various mechanisms for the antioxidant potential of *E. angustifolium* extracts, such as its protective role on stress-induced fibroblast senescence after exposure to ultraviolet (UV) radiation, this effect having a major contribution in skin’s photoprotection [13]. Karakurt et al. (2013) evaluated the antioxidant activity of *E. hirsutum* in vivo in a preclinical study performed on rats and showed a statistically significant increase in the activity of antioxidant enzymes (NADPH quinine oxireductase 1 and glutathione peroxidase), but the lack of extract standardization hinders discussion of the results, since the correlation with active compounds is not well defined [59]. So far, numerous studies determined the free radical scavenging activity in cell-free systems, but only a few took into consideration the effect of *Epilobium* extracts on ROS production in cellular models; this fact highlights the importance of the obtained results. Previous studies on the antioxidant capacity of willow herb extracts were correlated with the total phenolic content determination, and in a few cases, the extracts were characterized phytochemically in detail [1]. 

#### 3.8.3. Anti-Inflammatory Potential

**Figure 7:**
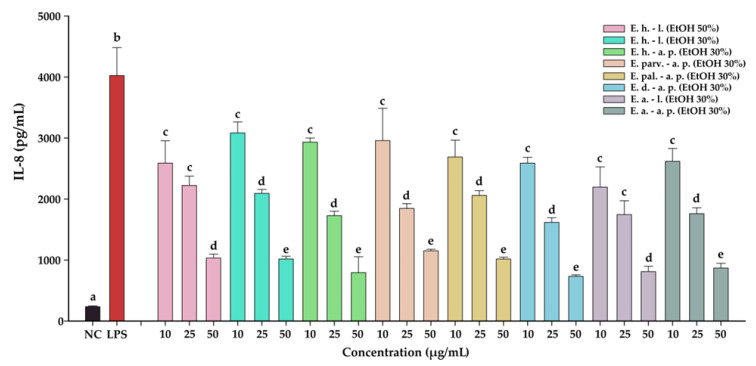
The anti-inflammatory potential of *E. hirsutum* leaves EtOH 50% (E. h.–l.), *E. hirsutum* leaves EtOH 30% (E. h.–l.), *E. hirsutum* aerial parts EtOH 30% (E. h.–a.p.), *E. parviflorum* aerial parts EtOH 30% (E. parv.–a. p.), *E. palustre* aerial parts EtOH 30% (E. pal.–a. p.), *E. dodonaei* aerial parts EtOH 30% (E. d.–a. p.), *E. angustifolium* leaves EtOH 30% (E. a.–l.), and *E. angustifolium* aerial parts EtOH 30% (E. a.–a. p.). The quantity of IL-6 was measured from cellular supernatants after a 24 h exposure to the extracts (10, 25, and 50 µg/mL) in combination with 100 ng/mL LPS. Data are expressed as mean ± standard deviation of three biological replicates. Different lowercase letters for bars representing the same extract and shown in identical colors indicate statistically significant differences in measured cytokine amounts (ANOVA + Holm–Sidak post hoc test at *p < 0.05* level of significance).

**Figure 8:**
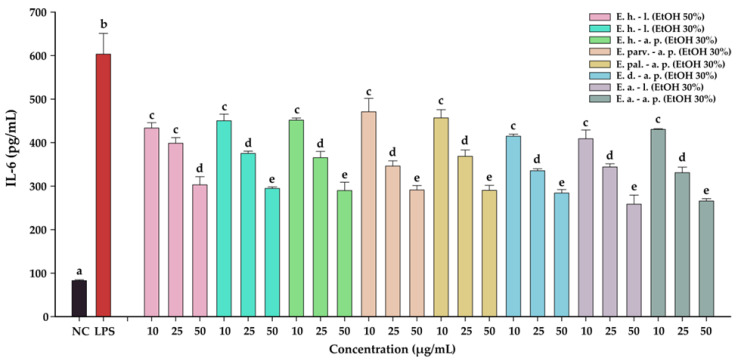
The anti-inflammatory potential of *E. hirsutum* leaves EtOH 50% (E. h.–l.), *E. hirsutum* leaves EtOH 30% (E. h.–l.), *E. hirsutum* aerial parts EtOH 30% (E. h.–a.p.), *E. parviflorum* aerial parts EtOH 30% (E. parv.–a. p.), *E. palustre* aerial parts EtOH 30% (E. pal.–a. p.), *E. dodonaei* aerial parts EtOH 30% (E. d.–a. p.), *E. angustifolium* leaves EtOH 30% (E. a.–l.), and *E. angustifolium* aerial parts EtOH 30% (E. a.–a. p.). The quantity of IL-8 was measured from cellular supernatants after a 24 h exposure to the extracts (10, 25, and 50 µg/mL) in combination with 100 ng/mL LPS. Data are expressed as mean ± standard deviation of three biological replicates. Different lowercase letters for bars representing the same extract and shown in identical colors indicate statistically significant differences in measured cytokine amounts (ANOVA + Holm–Sidak post hoc test at *p < 0.05* level of significance).

The anti-inflammatory effects of the *Epilobium* sp. extracts were evaluated by measuring the levels of two pro-inflammatory cytokines (IL-6, and IL-8) in an LPS inflammation-induced cellular model. The inflammation was induced in BJ cells by exposing the cells to lipopolysaccharides (LPS) isolated from *E. coli*. Exposure of BJ cells to 100 ng/mL LPS resulted in a robust pro-inflammatory response, the levels of IL-6 and IL-8 increasing approximately 7-times and 17-times, respectively (Table 10 and Table 11, Figure 7 and Figure 8, respectively). The anti-inflammatory activities of the eight extracts were further evaluated in this inflammation model by co-incubating for 24 h the cells with the extracts at non-cytotoxic doses and LPS. 

All eight extracts displayed anti-inflammatory effects, effects that were in almost all cases dose-dependent. All extracts presented anti-inflammatory properties starting from the lowest dose of 10 µg/mL, decreasing the level of IL-6 and IL-8 by approximately 25%. This effect was accentuated by the exposure dose: at the highest tested dose, the extracts decreased the levels of the two cytokines by approximately 50% in the case of IL-6, and 75% in the case of IL-8. Besides the dose-dependency of this effect, we evaluated the difference between the extracts. A Two-Way ANOVA analysis with the dose and the extract type as variables and the levels of the two cytokines as the measurement outcome was performed. Data analysis indicated that there are differences between the potencies of the extracts (*p <* 0.001); however, pairwise comparisons indicated statistical differences only in half of the comparisons. Based on the IL-6 and IL-8 levels, *E. dodonaei* aerial parts EtOH 30% and *E. angustifolium* leaves EtOH 30% extracts displayed the highest anti-inflammatory potential.

Previous studies evaluated the capacity of *Epilobium* sp. to ameliorate inflammatory response in various in vitro models. Kiss et al. (2011) investigated the role of oenothein B in relation to the antioxidant activity of *E. hirsutum*, *E. parviflorum*, and *E. angustifolium* and concluded that extracts inhibited the activity of lipoxygenase (IC_50_ around 25 mu g/mL) and inhibited the release of myeloperoxidase release from stimulated neutrophils [45]. Jung et al. (2021) evaluated the potential of *E. pyrriocholophum* hydroalcoholic extract to exert an anti-inflammatory effect in a chronic obstructive pulmonary disease model. The results showed that extracts of this species are capable of suppressing elevated IL-6 and IL-8 mRNA in a concentration-dependent manner [60]. Gunes at al. (2021) investigated the effects of *E. hirsutum* aerial parts and roots on human prostate cancer PC3 cell line. Their findings showed that extracts of this species decreased the gene expression of IL-8 and NF-κB but increased the gene expression of IL-6 [21]. A possible explanation could be that this cytokine can display both anti-inflammatory and pro-inflammatory effects, depending on experimental condition and tissue [61]. 

To the best of our knowledge, this was the first study to explore the anti-inflammatory effect of *E. hirsutum*, *E. parviflorum*, *E. palustre*, *E. dodonaei*, and *E. angustifolium* in an LPS inflammation-induced cellular model by direct measurement of IL-6 and IL-8 levels. The current results support the use of *Epilobium* species in BPH, prostatitis, bladder, kidney, and urinary tract diseases, pathologies which are commonly associated with inflammatory processes. 

## 4. Conclusions

To identify new sources of bioactive principles, the study of the chemical composition of *Epilobium* species that grow in Romania is important because of the broad spectrum of their medicinal properties and current use worldwide. The lack of any information concerning several indigenous plants (*E. palustre* and *E. dodonaei*) required the initiation of this complex research. Thus, the phytochemical and biological activities research of five *Epilobium* sp. harvested from Romanian flora was performed. New extraction techniques were performed on distinct plant parts, this aspect emphasizing the novelty of the presented study. The current research provides a scientific basis for the alleged medicinal properties of Romanian willow herb species, such as antimicrobial activity assessed in vitro on various microorganism (bacteria and fungi), antioxidant, anti-inflammatory, and antiproliferative effects respectively, evaluated on a healthy and cancerous cell line.

## Figures and Tables

**Table 1:** Details regarding the harvesting period and location of endemic *Epilobium* species.

Harvested Species	Location	GPS Position
*E. hirsutum* L.	Suceava, county Suceava, Romania	47.63266741707033, 26.247636499023365
*E. parviflorum* Schreb.		47.63266741707033, 26.247636499023365
*E. palustre* L.		47.63137600746175, 26.24739884699341
*E. dodonaei* Vill.	Pojorȃta, county Suceava, Romania	47.480482169195305, 25.489928143644686
*E. angustifolium* L.		47.48107622418807, 25.490536834857494

**Table 2:** Independent and dependent variables of the experimental design applied to find the optimum extraction conditions for *Epilobium* species extracts.

Variables	Level			
	−1	0		1
Independent variables (factors)				
Ethanol in solvent (%, *v/v)* (X_1_)	30	50		70
Extraction method (X_2_)	Ultra-turrax-assisted extraction (UTE)	Ultrasonic-assisted extraction (USE)	Maceration + UTE	Maceration + USE
Extraction time (min) (X_3_)	2	5		8
Dependent variables (responses)				
Total phenolic content (TPC, mg GAE/mL plant extract ^1^) (Y_1_)				
Total flavonoid content (TFC, mM QAE/mL plant extract ^2^) (Y_2_)				
Total antioxidant activity (TAA, mM TE/L plant extract ^3^) (Y_3_)				

^1^-mg GAE/mL plant extract = mg gallic acid equivalents/mL of plant extract; ^2^-mM QAE/mL plant extract = mM quercetin equivalents per mL of plant extract; ^3^-mM TE/L plant extract = mM Trolox equivalent per L plant extract.

**Table 3:** Matrix of the experimental design for the screening step and the results of total phenolic content, total flavonoid content, and total antioxidant activity for the extracts of *E. hirsutum* leaves.

Sample Code	Run Order	Factorial Design with Coded Values			Determination (Experimental Results)		
		X_1_	X_2_	X_3_	Y_1_ (TPC)	Y_2_ (TFC)	Y_3_ (TAA)
N1	6	30	A	2	3.003 ± 0.044	2.019 ± 0.087	66.364 ± 1.002
N2	15	70	A	2	2.789 ± 0.030	2.765 ± 0.058	56.263 ± 1.157
N3	4	30	B	2	2.726 ± 0.053	1.343 ± 0.034	61.566 ± 0.953
N4	10	70	B	2	2.220 ± 0.020	2.668 ± 0.060	48.813 ± 1.907
N5	1	30	C	2	2.676 ± 0.029	1.503 ± 0.041	62.197 ± 1.366
N6	14	70	C	2	2.635 ± 0.047	3.068 ± 0.041	43.131 ± 0.875
N7	2	30	D	2	2.072 ± 0.109	1.316 ± 0.073	51.338 ± 0.219
N8	19	70	D	2	2.544 ± 0.072	3.281 ± 0.063	42.374 ± 1.434
N9	12	30	A	8	3.321 ± 0.057	2.543 ± 0.063	65.101 ± 1.093
N10	7	70	A	8	3.145 ± 0.085	3.072 ± 0.027	59.924 ± 1.736
N11	5	30	B	8	2.997 ± 0.137	1.836 ± 0.054	59.167 ± 0.656
N12	17	70	B	8	2.440 ± 0.089	2.690 ± 0.015	40.732 ± 1.434
N13	3	30	C	8	3.415 ± 0.096	1.965 ± 0.035	69.141 ± 1.434
N14	9	70	C	8	2.314 ± 0.114	3.303 ± 0.068	65.354 ± 0.579
N15	8	30	D	8	2.682 ± 0.086	1.445 ± 0.074	62.702 ± 1.907
N16	16	70	D	8	2.443 ± 0.127	3.028 ± 0.020	42.374 ± 1.157
N17	13	50	A	5	3.447 ± 0.175	2.552 ± 0.027	66.490 ± 0.953
N18	18	50	A	5	2.972 ± 0.077	2.241 ± 0.034	64.217 ± 0.579
N19	11	50	A	5	3.019 ± 0.311	2.201 ± 0.028	66.237 ± 1.093

X_1_—ethanol ratio (%, *v/v*); X_2_—extraction methods: A—ultra-turrax extraction (UTE); B—ultrasonic-assisted extraction (USE); C—maceration (48 h) followed by UTE; D—maceration (48 h) followed by USE. X_3_—extraction time (min). Y_1_—total polyphenolic content (TPC), expressed as mg gallic acid equivalent (GAE)/mL extract. Y_2_—total flavonoid content (TFC), expressed as mg rutin equivalent/mL extract. Y_3_—total antioxidant activity (TAA) through TEAC assay, expressed as mM Trolox equivalent (TE)/L extract.

**Table 4:** The fitting parameters for the evaluated output variables.

Quantifiable Responses	R^2^	R^2^ Adjusted	Q^2^	SDY	RSD	N	Model Validity	Reproducibility
Total phenolic content (Y_1_)	0.848	0.544	0.390	0.396	0.267	19	0.843	0.563
Total flavonoid content (Y_2_)	0.962	0.888	0.362	0.663	0.221	19	0.793	0.916
Total antioxidant activity (Y_3_)	0.888	0.665	0.338	9.632	5.567	19	0.146	0.983

**Table 5:** Determination of TPC, TFC, and antioxidant activity through DPPH and TEAC assays for the optimized *Epilobium* sp. extracts (mean value ± SD, n = 3).

No	Species	Plant Part	Solvent Mixture	TPC ^1^	TFC ^2^	DPPH ^3^	TEAC ^4^
1	*E. hirsutum*	leaves	EtOH 50%	4.201 ± 0.085	3.779 ± 0.040	13.051 ± 0.161	75.404 ± 11.347
2	*E. hirsutum*	leaves	EtOH 30%	3.811 ± 0.132	3.148 ± 0.043	12.875 ± 0.265	73.636 ± 6.201
3	*E. hirsutum*	aerial parts	EtOH 30%	3.786 ± 0.066	1.583 ± 0.034	12.279 ± 0.418	55.707 ± 0.875
4	*E. hirsutum*	roots	EtOH 30%	1.497 ± 0.095	-	6.227 ± 0.211	33.99 ± 4.172
5	*E. parviflorum*	aerial parts	EtOH 30%	5.025 ± 0.139	2.996 ± 0.043	12.261 ± 0.548	89.545 ± 6.733
6	*E. palustre*	aerial parts	EtOH 30%	3.025 ± 0.029	1.508 ± 0.015	12.770 ± 0.399	54.192 ± 6.443
7	*E. dodonaei*	aerial parts	EtOH 30%	6.528 ± 0.118	2.419 ± 0.013	13.612 ± 0.169	91.818 ± 2.731
8	*E. angustifolium*	leaves	EtOH 30%	4.805 ± 0.058	2.681 ± 0.147	13.525 ± 0.342	81.97 ± 0.758
9	*E. angustifolium*	aerial parts	EtOH 30%	4.472 ± 0.086	1.285 ± 0.127	13.63 ± 0.213	86.768 ± 7.039

^1^ TPC—total polyphenolic content, expressed as mg gallic acid equivalent (GAE)/mL extract; ^2^ TFC—total flavonoid content, expressed as mg rutin equivalent/mL extract; ^3^ DPPH—antioxidant activity through DPPH assay, expressed as mg quercetin equivalent (QE)/mL extract; ^4^ TEAC—antioxidant potential through TEAC assay, expressed as mM Trolox equivalent (TE)/L extract.

**Table 6:** Identification and Quantification of Bioactive Compounds by LC/MS.

Plant Species		*E. hirsutum*	*E. hirsutum*	*E. hirsutum*	*E. hirsutum*	*E. parviflorum*	*E. palustre*	*E. dodonaei*	*E.* *angustifolium*	*E. angustifolium*
Plant Part		Leaves	Leaves	Aerial Parts	Roots	Aerial Parts	Aerial Parts	Aerial Parts	Leaves	Aerial Parts
Solvent		EtOH 50%	EtOH 30%	EtOH 30%	EtOH 30%	EtOH 30%	EtOH 30%	EtOH 30%	EtOH 30%	EtOH 30%
Polyphenols (μg/g d.w.)	Caftaric acid	458.82 ± 26.05	468.31 ± 26.28	311.68 ± 18.89	13.44 ± 0.67	1002.29 ± 57.94	1017.32 ± 58.17	636.02 ± 38.04	136.06 ± 7.11	12.65 ± 0.91
	Gentisic acid	-	6.08 ± 0.26	4.68 ± 0.31	-	<LOQ	<LOQ	<LOQ	<LOQ	<LOQ
	Caffeic acid	<LOQ	<LOQ	<LOQ	-	<LOQ	<LOQ	<LOQ	<LOQ	<LOQ
	Chlorogenic acid	<LOQ	<LOQ	<LOQ	<LOQ	<LOQ	<LOQ	<LOQ	395.83 ± 25.15	508.31 ± 31.24
	*p*-coumaric acid	36.91 ± 2.17	28.48 ± 1.63	20.66 ± 1.10	-	18.25 ± 0.95	10.43 ± 0.44	16.45 ± 0.87	9.83 ± 0.56	<LOQ
	Ferulic acid	-	-	-	-	-	<LOQ	-	-	-
	Hyperoside	424.89 ± 22.98	308.79 ± 16.24	199.95 ± 11.07	-	180.26 ± 13.33	89.04 ± 4.68	69.34 ± 4.51	<LOQ	<LOQ
	Isoquercitrin	<LOQ	<LOQ	<LOQ	-	<LOQ	<LOQ	<LOQ	225.42 ± 10.93	265.49 ± 17.56
	Myricetin	199.41 ± 10.39	171.11 ± 8.84	116.05 ± 5.55	-	54.10 ± 3.11	36.50 ± 2.26	56.39 ± 3.62	<LOQ	<LOQ
	Quercitrin	540.22 ± 22.96	383.17 ± 25.20	233.61 ± 13.87	7.39 ± 0.48	487.87 ± 31.16	173.78 ± 11.04	594.44 ± 43.61	285.96 ± 17.52	783.27 ± 54.03
	Quercetol	30.92 ± 1.67	29.26 ± 1.78	21.01 ± 1.48	-	11.10 ± 0.61	8.34 ± 0.56	<LOQ	13.30 ± 0.61	14.40 ± 0.82
	Kaempferol-3-rhamnoside	34.33 ± 2.24	29.63 ± 1.70	15.51 ± 0.98	-	76.68 ± 4.63	32.77 ± 1.74	705.60 ± 50.09	202.15 ± 11.18	279.01 ± 16.11
	Kaempferol	9.47 ± 0.66	18.09 ± 1.07	13.45 ± 0.86	-	7.48 ± 0.54	7.48 ± 0.42	8.80 ± 0.48	4.16 ± 0.22	3.50 ± 0.21
	Epicatechin	0.45 ± 0.03	-	1.31 ± 0.07	-	0.79 ± 0.03	-	-	-	-
	Catechin	3.17 ± 0.23	2.56 ± 0.16	3.33 ± 0.21	1.02 ± 0.05	9.21 ± 0.44	4.83 ± 0.25	1.33 ± 0.07	-	-
	Gallic acid	285.35 ± 18.64	377.58 ± 27.44	349.93 ± 17.17	47.94 ± 2.28	227.13 ± 12.46	308.18 ± 20.96	662.28 ± 42.46	426.41 ± 23.04	409.17 ± 28.36
	Proto-catechuic acid	0.59 ± 0.02	0.99 ± 0.05	0.75 ± 0.04	-	4.95 ± 0.27	5.68 ± 0.27	6.60 ± 0.41	7.21 ± 0.38	11.39 ± 0.63
Sterols (ng/g d.w.)	Ergosterol	-	-	1.14 ± 0.06	1.99 ± 0.13	1.06 ± 0.06	8.91 ± 0.63	6.54 ± 0.39	4.26 ± 0.25	3.19 ± 0.18
	Beta-Sitosterol	-	1206.29 ± 80.12	1536.74 ± 77.24	1407.06 ± 110.59	1590.50 ± 112.11	1664.74 ± 98.05	2044.87 ± 164.58	1957.09 ± 93.99	2694.80 ± 152.71
	Campesterol	-	<LOQ	3.04 ± 0.17	3.39 ± 0.17	2.67 ± 0.15	7.45 ± 0.31	4.95 ± 0.19	1.98 ± 0.12	4.95 ± 0.26
Tocopherols (ng/g d.w.)	α-tocopherol	449.97 ± 27.81	8633.68 ± 356.97	6527.96 ± 453.25	446.17 ± 21.82	9435.45 ± 398.51	3141.47 ± 174.18	5730.48 ± 424.54	6463.38 ± 332.45	6079.84 ± 400.13
	γ-tocopherol	-	810.09 ± 45.48	2558.96 ± 139.44	-	7796.07 ± 421.07	3095.59 ± 192.49	1264.03 ± 52.52	529.72 ± 31.45	734.35 ± 48.05
	δ-tocopherol	8.59 ± 0.41	73.64 ± 3.51	331.71 ± 20.94	8.59 ± 0.46	572.76 ± 33.14	256.26 ± 16.95	105.32 ± 6.36	48.37 ± 2.93	41.75 ± 2.01
Ellagitannin (mg/g d.w.)	Oenothein B	70.81 ± 4.03	54.92 ± 3.57	73.49 ± 3.89	-	98.84 ± 5.89	41.88 ± 2.91	106.82 ± 7.45	54.78 ± 3.21	51.82 ± 2.93

Not found; <LOQ—identified based on MS spectra but not determined quantitatively, below limit of quantification. Data are expressed as mean ± SD (n = 3).

**Table 7:** The results of the in vitro qualitative screening—the disk diffusion test.

Micro-Organism Strain	Inhibition Area Diameter of Plant Extracts Versus Standard Drugs (Expressed in mm)										
	1	2	3	4	5	6	7	8	9	A	M
*Staphylococcus aureus*	12.22	12.51	9.15	6.94	10.83	8.57	12.45	10.73	8.84	24.38	-
*Enterococcus fecalis*	9.65	6.62	9.1	7.35	8.56	8.2	9.52	8.43	7.88	16.8	-
*Listeria monocytogenes*	8.05	7.71	9.86	7.11	10.29	8.29	10.48	8.82	8.23	18.96	-
*Bacillus cereus*	14.09	14.61	14.98	9.16	15.44	11.95	15.42	13.83	14.13	8.83	-
*Candida albicans*	10.92	9.82	11.88	10.91	12.17	10.65	10.54	9.97	11.71	-	14.46

Legend: 1—*E. hirsutum* leaves (EtOH 50%), 2—*E. hirsutum* leaves (EtOH 30%), 3—*E. hirsutum* aerial parts (EtOH 30%), 4—*E. hirsutum* roots (EtOH 30%), 5—*E. parviflorum* aerial parts (EtOH 30%), 6—*E. palustre* aerial parts (EtOH 30%), 7—*E. dodonaei* aerial parts (EtOH 30%), 8—*E. angustifolium* leaves (EtOH 30%), 9—*E. angustifolium* aerial parts (EtOH 30%), A—amoxicillin, M—miconazole.

**Table 8:** The results of the in vitro quantitative evaluation—MIC test.

Micro-Organism Strain	Plant Extracts Activity Versus Ethanol and Their Corresponding Minimum Inhibitory Concentrations																			
	1		2		3		4		5		6		7		8		9		E	
	MIC 100	MIC 50	MIC 100	MIC 50	MIC 100	MIC 50	MIC 100	MIC 50	MIC 100	MIC 50	MIC 100	MIC 50	MIC 100	MIC 50	MIC 100	MIC 50	MIC 100	MIC 50	30% MIC 100	50% MIC 100
*Staphylococcus aureus*	1/ 64	1/ 128	1/ 64	1/ 128	1/ 64	1/ 128	1/ 32	1/ 64	1/ 64	1/ 128	1/ 64	1/ 128	1/ 64	1/ 128	1/ 64	1/ 128	1/ 64	1/ 64	0	0
*Enterococcus faecalis*	1/ 128	1/ 256	1/ 128	1/ 256	1/ 128	1/ 256	1/ 128	1/ 256	1/ 128	1/ 256	1/ 128	1/ 256	1/ 128	1/ 256	1/ 64	1/ 128	1/ 64	1/ 64	0	0
*Listeria monocytogenes*	1/ 64	1/ 128	1/ 64	1/ 128	1/ 64	1/ 64	1/ 64	1/ 128	1/ 64	1/ 128	1/ 64	1/ 128	1/ 128	1/ 128	1/ 64	1/ 128	1/ 32	1/ 64	0	0
*Bacillus cereus*	1/ 64	1/ 128	1/ 64	1/ 128	1/ 128	1/ 256	1/ 64	1/ 128	1/ 128	1/ 128	1/ 64	1/ 64	1/ 64	1/ 128	1/ 64	1/ 128	1/ 64	1/ 64	0	0
*Candida albicans*	1/ 128	1/ 256	1/ 64	1/ 128	1/ 128	1/ 256	1/ 64	1/ 64	1/ 64	1/ 128	1/ 32	1/ 64	1/ 64	1/ 128	1/ 32	1/ 64	1/ 32	1/ 32	0	0

Legend: MIC—minimum inhibitory concentration; 1—*E. hirsutum* leaves (EtOH 50%), 2—*E. hirsutum* leaves (EtOH 30%), 3—*E. hirsutum* aerial parts (EtOH 30%), 4—*E. hirsutum* roots (EtOH 30%), 5—*E. parviflorum* aerial parts (EtOH 30%), 6—*E. palustre* aerial parts (EtOH 30%), 7—*E. dodonaei* aerial parts (EtOH 30%), 8—*E. angustifolium* leaves (EtOH 30%), 9—*E. angustifolium* aerial parts (EtOH 30%), E—ethanol.

**Table 9:** IC_50_ values (µg/mL) after exposure of human prostate cancer cells (LNCaP) and human normal foreskin fibroblasts (BJ) to the *Epilobium* sp. extracts for 24 h. The ratio between the IC_50_s for the cancerous and normal cells obtained was also calculated for selectivity approximation.

Cell Line	IC_50_ (µg/mL)							
	1	2	3	4	5	6	7	8
LNCaP	32.94	30.11	20.18	37.96	32.42	18.38	35.48	27.33
BJ	201.00	201.69	183.70	322.65	189.44	126.05	250.24	288.68
Ratio of IC_50_	6.10	6.69	9.10	8.49	5.84	6.85	7.05	10.56

Legend: 1—*E. hirsutum* leaves (EtOH 50%), 2—*E. hirsutum* leaves (EtOH 30%), 3—*E. hirsutum* aerial parts (EtOH 30%), 4—*E. parviflorum* aerial parts (EtOH 30%), 5—*E. palustre* aerial parts (EtOH 30%), 6—*E. dodonaei* aerial parts (EtOH 30%), 7—*E. angustifolium* leaves (EtOH 30%), 8—*E. angustifolium* aerial parts (EtOH 30%).

**Table 10:** The anti-inflammatory potential of *Epilobium* sp. extracts. The quantity of IL-6 was measured from cellular supernatants after a 24 h exposure to the extracts (10, 25, and 50 µg/mL) in combination with 100 ng/mL LPS. Data are expressed as mean ± standard deviation of three biological replicates.

Extract (E)	IL-6 (pg/mL)				
	LPS + 10 µg/mL E	LPS + 25 µg/mL E	LPS + 50 µg/mL E	NC	LPS
1	433 ± 12	398 ± 13	303 ± 19	82 ± 2	603 ± 47
2	450 ± 15	375 ± 5	295 ± 3		
3	451 ± 5	365 ± 15	289 ± 19		
4	471 ± 31	346 ± 12	291 ± 10		
5	456 ± 19	368 ± 15	290 ± 12		
6	415 ± 5	335 ± 4	284 ± 8		
7	409 ± 20	344 ± 7	258 ± 21		
8	430 ± 2	331 ± 13	266 ± 5		

Legend: 1—*E. hirsutum* leaves (EtOH 50%), 2—*E. hirsutum* leaves (EtOH 30%), 3—*E. hirsutum* aerial parts (EtOH 30%), 4—*E. parviflorum* aerial parts (EtOH 30%), 5—*E. palustre* aerial parts (EtOH 30%), 6—*E. dodonaei* aerial parts (EtOH 30%), 7—*E. angustifolium* leaves (EtOH 30%), 8—*E. angustifolium* aerial parts (EtOH 30%).

**Table 11:** The anti-inflammatory potential of *Epilobium* sp. extracts. The quantity of IL-8 was measured from cellular supernatants after a 24 h exposure to the extracts (10, 25, and 50 µg/mL) in combination with 100 ng/mL LPS. Data are expressed as mean ± standard deviation of three biological replicates.

Extract (E)	IL-8 (pg/mL)				
	LPS + 10 µg/mL E	LPS + 25 µg/mL E	LPS + 50 µg/mL E	NC	LPS
1	2588 ± 367	2221 ± 153	1033 ± 63	235 ± 12	4023 ± 458
2	3084 ± 178	2092 ± 65	1015 ± 46		
3	2932 ± 66	1727 ± 73	793 ± 259		
4	2958 ± 529	1844 ± 79	1150 ± 25		
5	2688 ± 275	2058 ± 79	1016 ± 30		
6	2587 ± 95	1617 ± 74	731 ± 25		
7	2194 ± 331	1744 ± 226	810 ± 86		
8	2618 ± 211	1758 ± 96	869 ± 77		

Legend: 1—*E. hirsutum* leaves (EtOH 50%), 2—*E. hirsutum* leaves (EtOH 30%), 3—*E. hirsutum* aerial parts (EtOH 30%), 4—*E. parviflorum* aerial parts (EtOH 30%), 5—*E. palustre* aerial parts (EtOH 30%), 6—*E. dodonaei* aerial parts (EtOH 30%), 7—*E. angustifolium* leaves (EtOH 30%), 8—*E. angustifolium* aerial parts (EtOH 30%).

## Data Availability

Data is contained within the article.

## Supplementary Materials

The following supporting information can be downloaded at https://www.mdpi.com/article/10.3390/antiox12010091/s1, Figure S1: Sample 1—*E. hirsutum* leaves (EtOH 50%)—The UV chromatogram for the individual polyphenolic compounds quantified by the first LC-MS method (A), the total ion chromatogram (TIC) for polyphenols analyzed with the second analytical method (B), and the TIC for tocopherols (C); Figure S2: Sample 2—*E. hirsutum* leaves (EtOH 30%)—The UV chromatogram for the individual polyphenolic compounds quantified by the first LC-MS method (A), the TIC for polyphenols analyzed with the second analytical method (B), and the TIC for tocopherols (C); Figure S3: Sample 3—*E. hirsutum* aerial parts (EtOH 30%)—The UV chromatogram for the individual polyphenolic compounds quantified by the first LC-MS method (A), the TIC for polyphenols analyzed with the second analytical method (B), and the TIC for tocopherols (C); Figure S4: Sample 4—*E. hirsutum* roots (EtOH 30%)—The UV chromatogram for the individual polyphenolic compounds quantified by the first LC-MS method (A), the TIC for polyphenols analyzed with the second analytical method (B), and the TIC for tocopherols (C); Figure S5: Sample 5—*E. parviflorum* aerial parts (EtOH 30%)—The UV chromatogram for the individual polyphenolic compounds quantified by the first LC-MS method (A), the TIC for polyphenols analyzed with the second analytical method (B), and the TIC for tocopherols (C); Figure S6: Sample 6—*E. palustre* aerial parts (EtOH 30%)—The UV chromatogram for the individual polyphenolic compounds quantified by the first LC-MS method (A), the TIC for polyphenols analyzed with the second analytical method (B), and the TIC for tocopherols (C); Figure S7: Sample 7—*E. dodonaei* aerial parts (EtOH 30%)—The UV chromatogram for the individual polyphenolic compounds quantified by the first LC-MS method (A), the TIC for polyphenols analyzed with the second analytical method (B), and the TIC for tocopherols (C); Figure S8: Sample 8—*E. angustifolium* leaves (EtOH 30%) - The UV chromatogram for the individual polyphenolic compounds quantified by the first LC-MS method (A), the TIC for polyphenols analyzed with the second analytical method (B), and the TIC for tocopherols (C); Figure S9: Sample 9—*E. angustifolium* aerial parts (EtOH 30%)—The UV chromatogram for the individual polyphenolic compounds quantified by the first LC-MS method (A), the TIC for polyphenols analyzed with the second analytical method (B), and the TIC for tocopherols (C).
