# Supplementary material for: Epilobium Species: From Optimization of the Extraction Process to Evaluation of Biological Properties
Source: Antioxidants (Basel). 2022 Dec 30;12(1):91. doi: 10.3390/antiox12010091 (PMC9854965; doi:10.3390/antiox12010091)
Supplement: Supplementary file 1 [file antioxidants-12-00091-s001.zip › antioxidants-2089177-supplementary.pdf]

Sample 1 – *E. hirsutum* leaves (EtOH 50%)

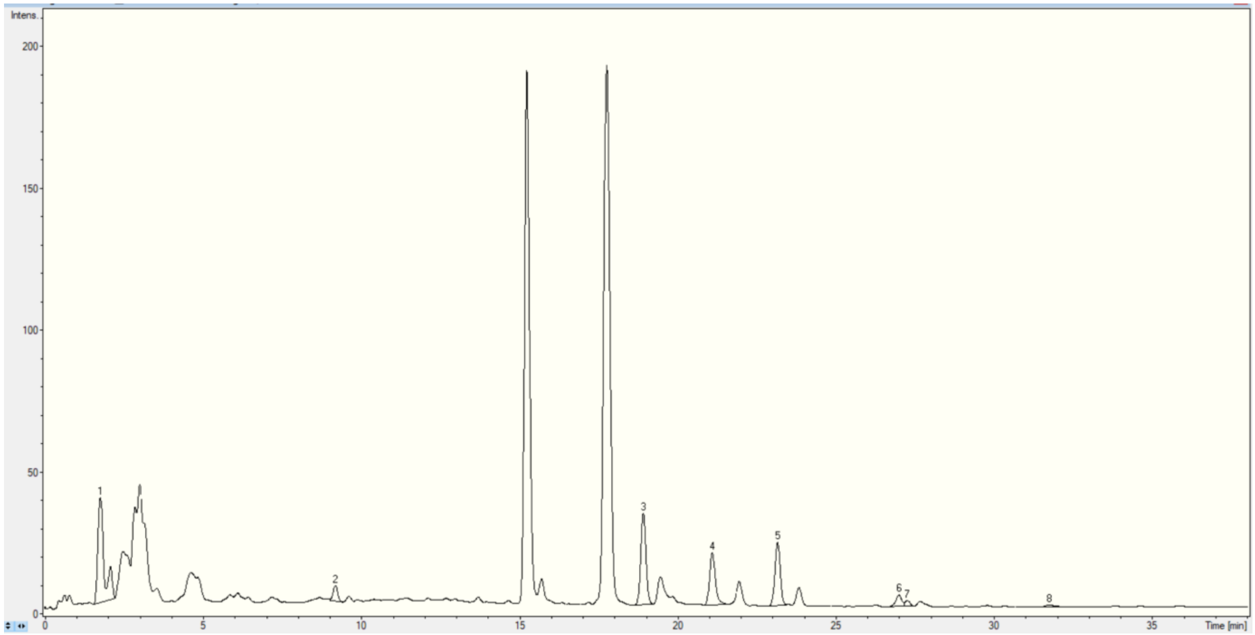

Figure S1 (A): The UV chromatogram for the individual polyphenolic compounds quantified by the first LC-MS method

Legend: 1- caftaric acid; 2- *p*-coumaric acid; 3- hyperoside; 4- myricetin; 5- quercitrin; 6- quercetol; 7- kaempferol-3-rhamnoside; 8- kaempferol.

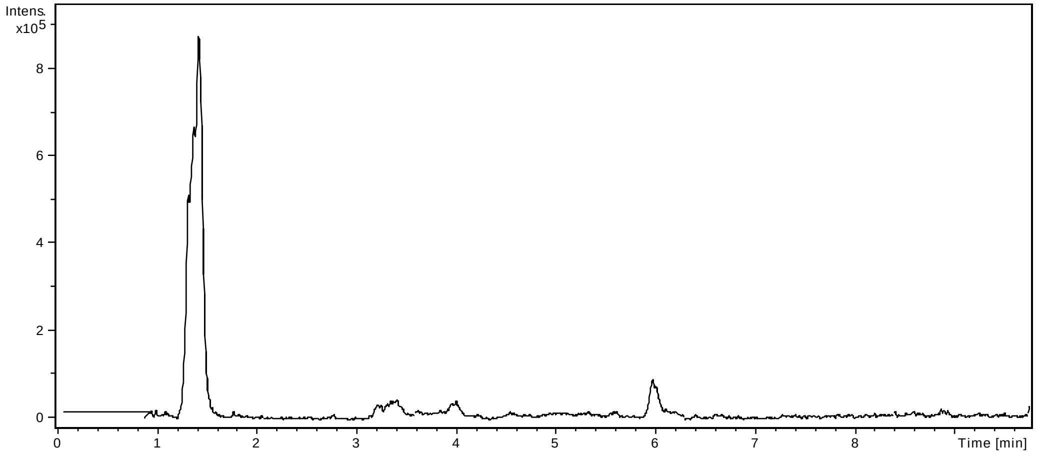

Figure S1 (B): The total ion chromatogram (TIC) for polyphenols analyzed with the second analytical method (Gallic acid – R_T=1.5 min; Protocatechuic acid – R_T=2.8 min; Catechin – R_T=6.0 min; Vanilic acid – R_T=6.7 min; Syringic acid – R_T=8.4 min; Epicatechin – R_T=9.0 min)

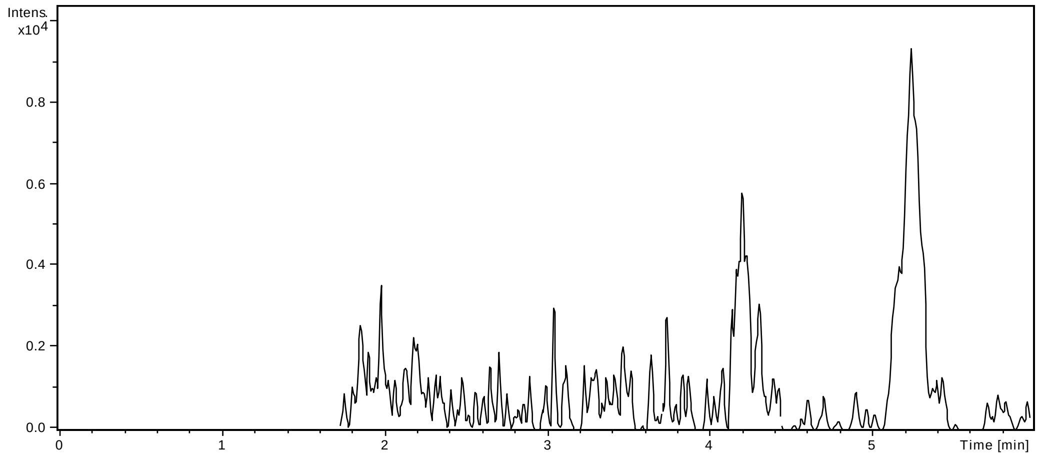

Figure S1 (C): The TIC for tocopherols (δ-tocopherol – R_T=3.3 min; γ-tocopherol – R_T=4.1 min; α-tocopherol – R_T=5.1 min)

Sample 2 – *E. hirsutum* leaves (EtOH 30%)

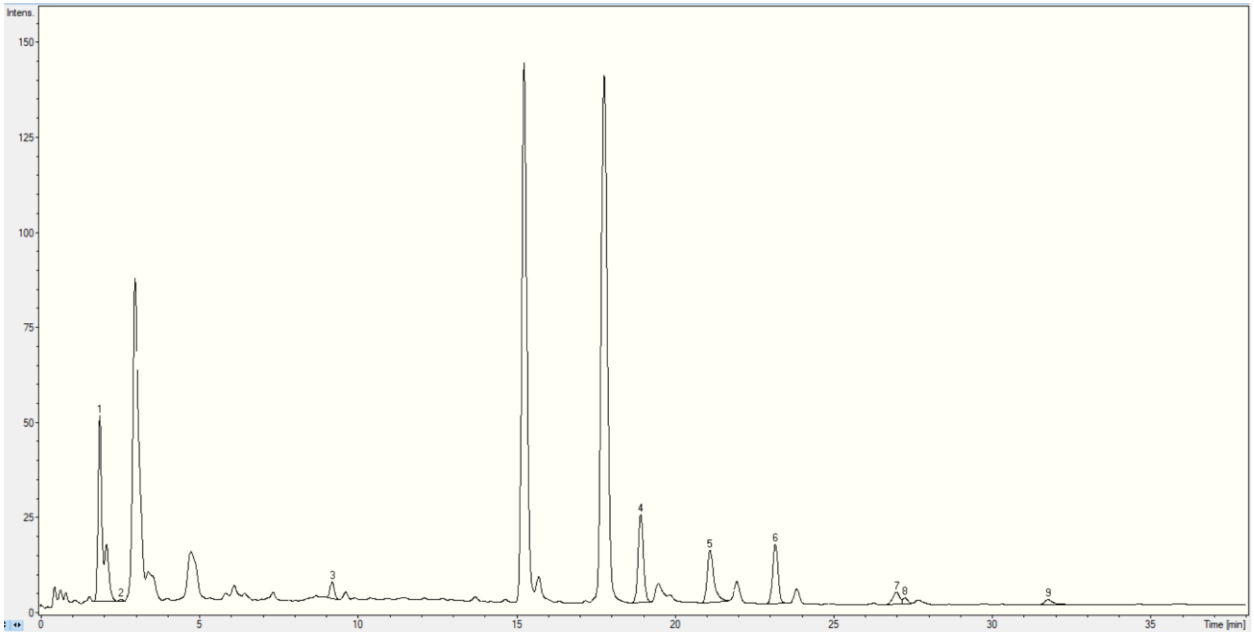

Figure S2 (A): The UV chromatogram for the individual polyphenolic compounds quantified by the first LC-MS method

Legend: 1- caftaric acid; 2- gentisic acid; 3- *p*-coumaric acid; 4- hyperoside; 5- myricetin; 6- quercitrin; 7- quercetol; 8- kaempferol-3-rhamnoside; 9- kaempferol.

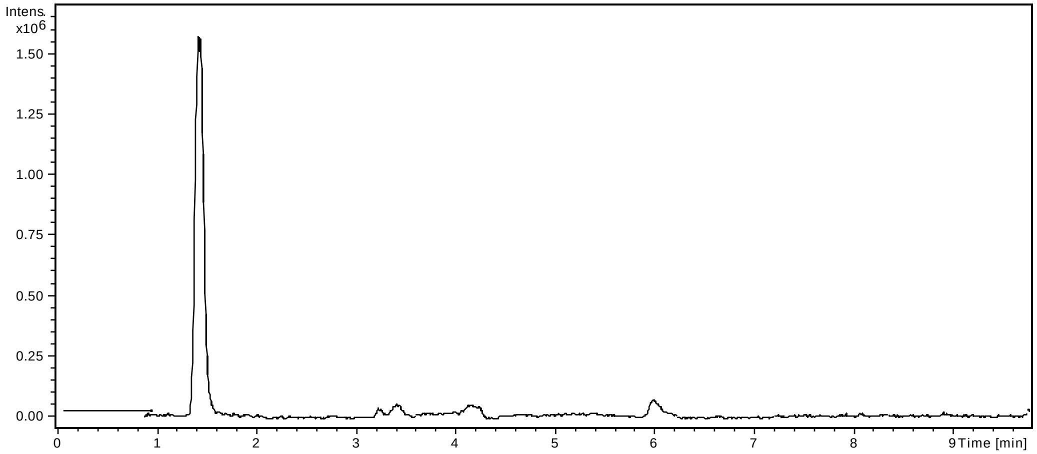

Figure S2 (B): The TIC for polyphenols analyzed with the second analytical method (Gallic acid – R_T=1.5 min; Protocatechuic acid – R_T=2.8 min; Catechin – R_T=6.0 min; Vanilic acid – R_T=6.7 min; Syringic acid – R_T=8.4 min; Epicatechin – R_T=9.0 min)

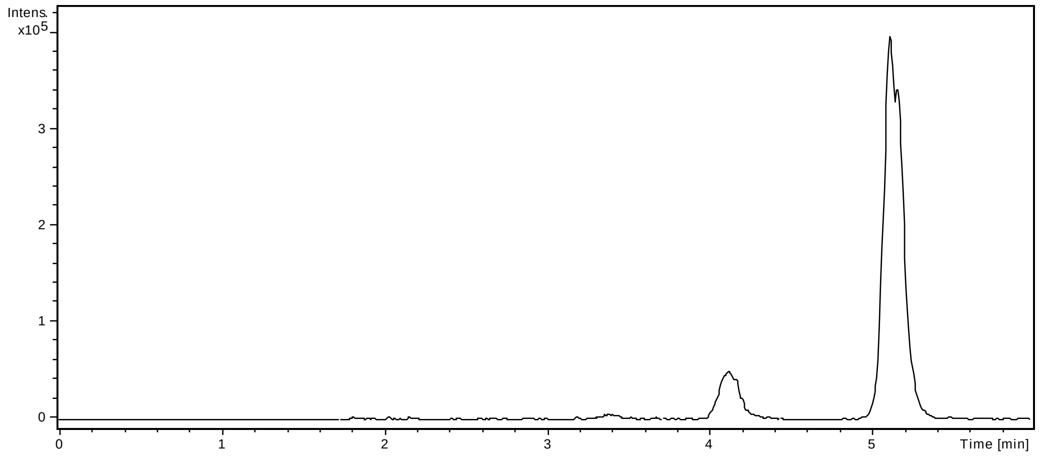

Figure S2 (C): TIC for tocopherols (δ-tocopherol – R_T=3.3 min; γ-tocopherol – R_T=4.1 min; α-tocopherol – R_T=5.1 min)

Sample 3 – *E. hirsutum* aerial parts (EtOH 30%)

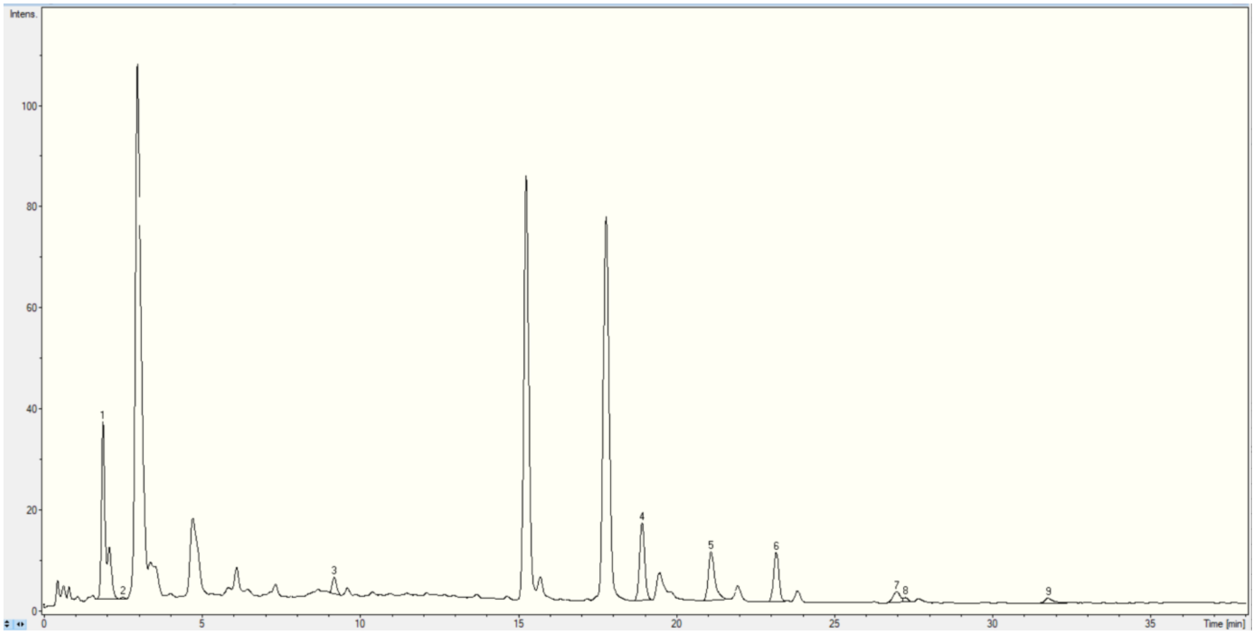

Figure S3 (A): The UV chromatogram for the individual polyphenolic compounds quantified by the first LC-MS method

Legend: 1- caftaric acid; 2- gentisic acid; 3- *p*-coumaric acid; 4- hyperoside; 5- myricetin; 6- quercitrin; 7- quercetol; 8- kaempferol-3-rhamnoside; 9- kaempferol.

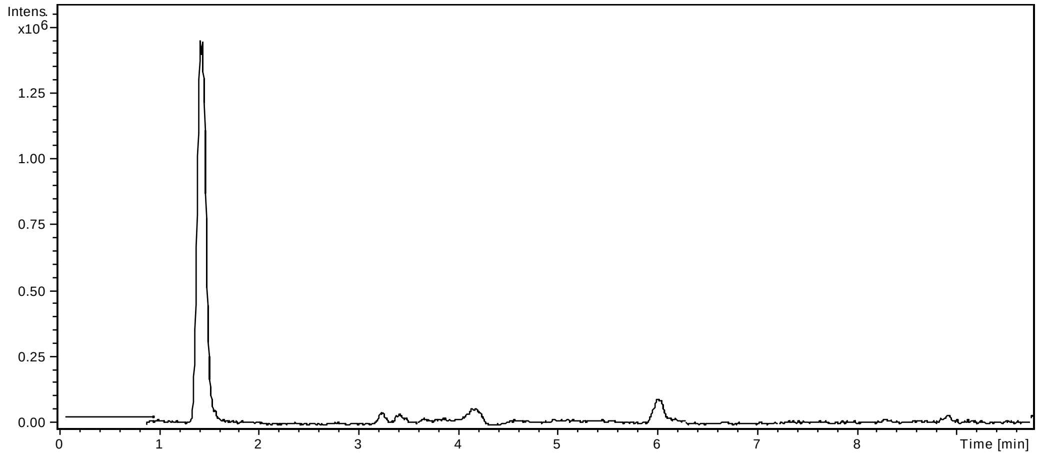

Figure S3 (B): The TIC for polyphenols analyzed with the second analytical method (Gallic acid – R_T=1.5 min; Protocatechuic acid – R_T=2.8 min; Catechin – R_T=6.0 min; Vanilic acid – R_T=6.7 min; Syringic acid – R_T=8.4 min; Epicatechin – R_T=9.0 min)

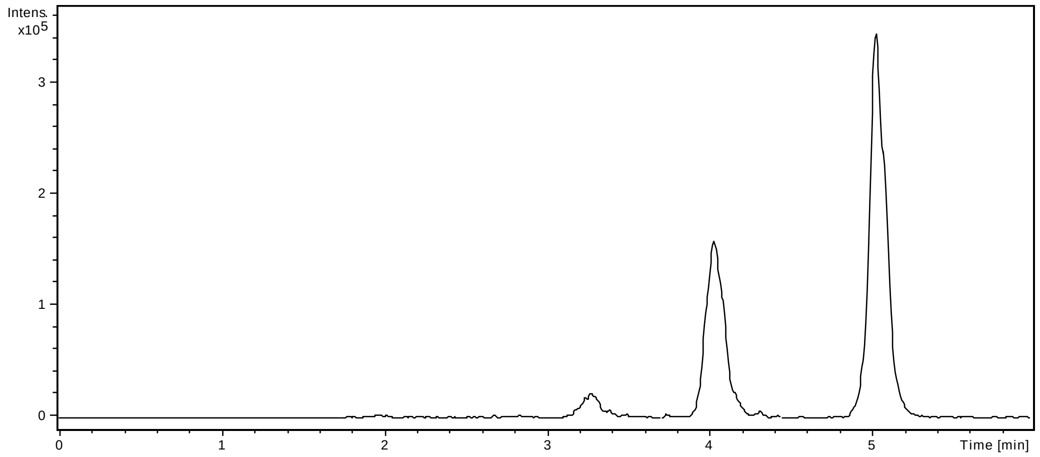

Figure S3 (C): TIC for tocopherols (δ-tocopherol – R_T=3.3 min; γ-tocopherol – R_T=4.1 min; α-tocopherol – R_T=5.1 min)

Sample 4 – *E. hirsutum* roots (EtOH 30%)

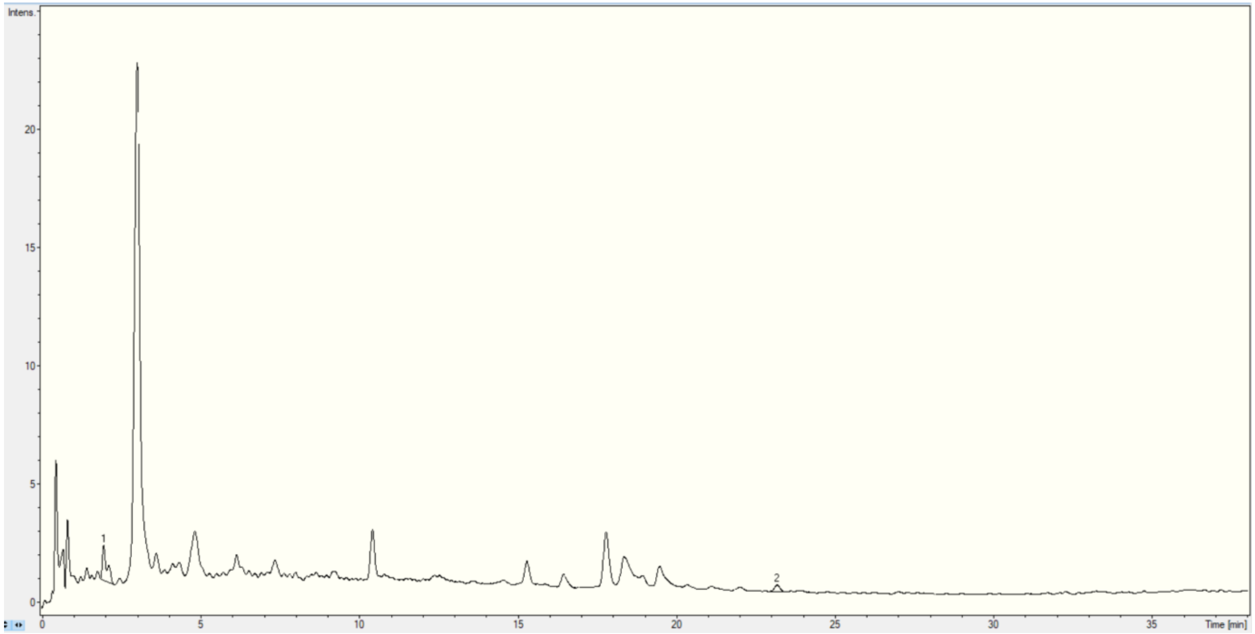

Figure S4 (A): The UV chromatogram for the individual polyphenolic compounds quantified by the first LC-MS method

Legend: 1- caftaric acid; 2- quercitrin.

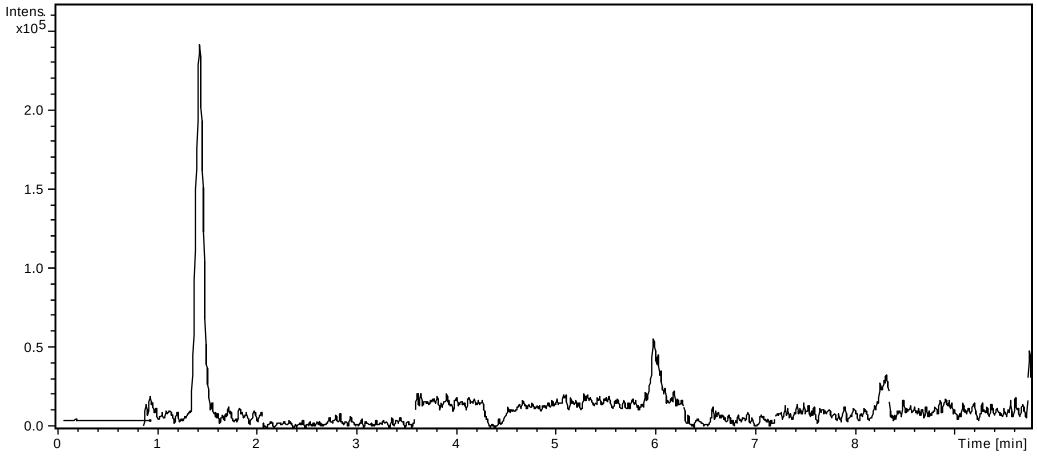

Figure S4 (B): The TIC for polyphenols analyzed with the second analytical method (Gallic acid – R_T=1.5 min; Protocatechuic acid – R_T=2.8 min; Catechin – R_T=6.0 min; Vanilic acid – R_T=6.7 min; Syringic acid – R_T=8.4 min; Epicatechin – R_T=9.0 min)

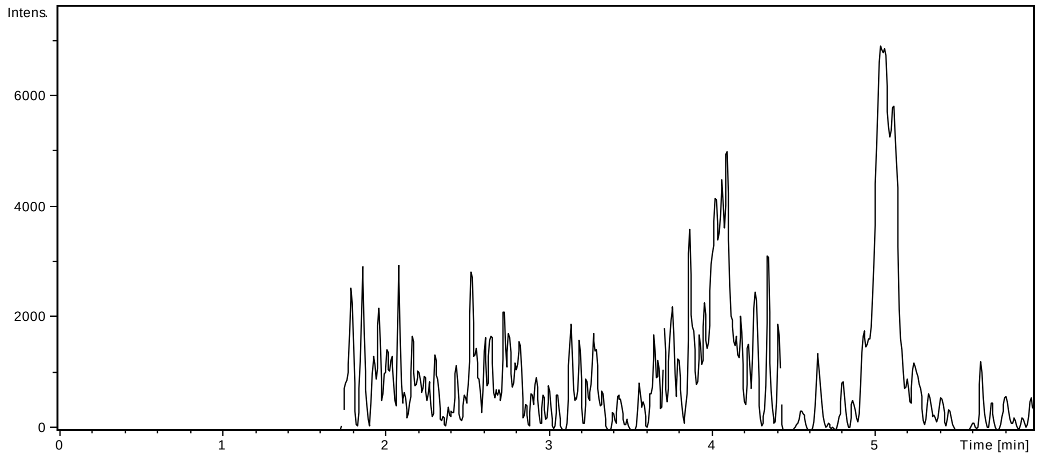

Figure S4 (C): TIC for tocopherols (δ-tocopherol – R_T=3.3 min; γ-tocopherol – R_T=4.1 min; α-tocopherol – R_T=5.1 min)

Sample 5 – *E. parviflorum* aerial parts (EtOH 30%)

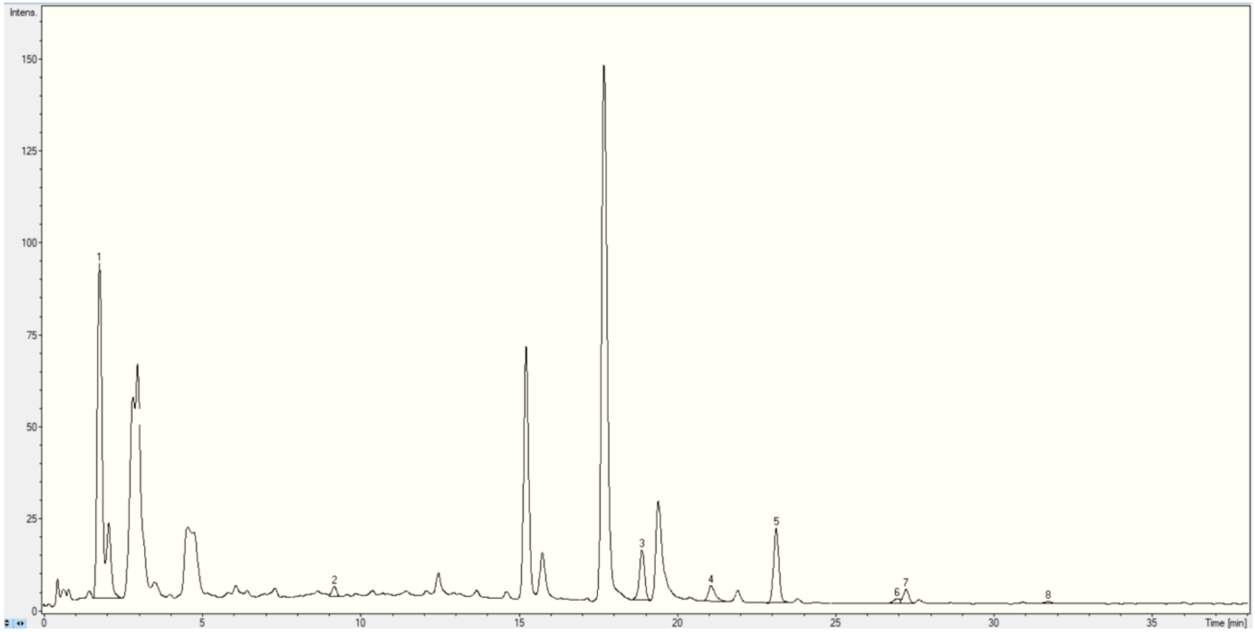

Figure S5 (A): The UV chromatogram for the individual polyphenolic compounds quantified by the first LC-MS method

Legend: 1- caftaric acid; 2- *p*-coumaric acid; 3- hyperoside; 4- myricetin; 5- quercitrin; 6- quercetol; 7- kaempferol-3-rhamnoside; 8- kaempferol.

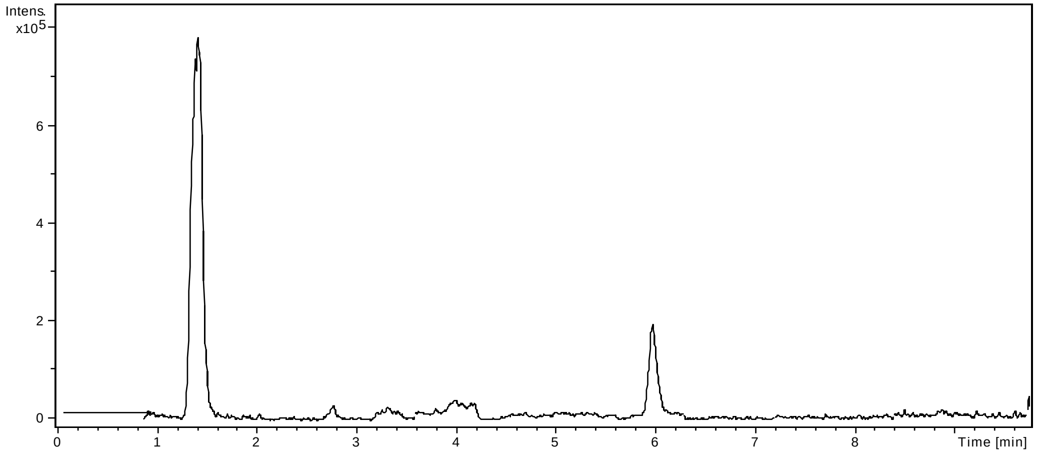

Figure S5 (B): The TIC for polyphenols analyzed with the second analytical method (Gallic acid – R_T=1.5 min; Protocatechuic acid – R_T=2.8 min; Catechin – R_T=6.0 min; Vanilic acid – R_T=6.7 min; Syringic acid – R_T=8.4 min; Epicatechin – R_T=9.0 min)

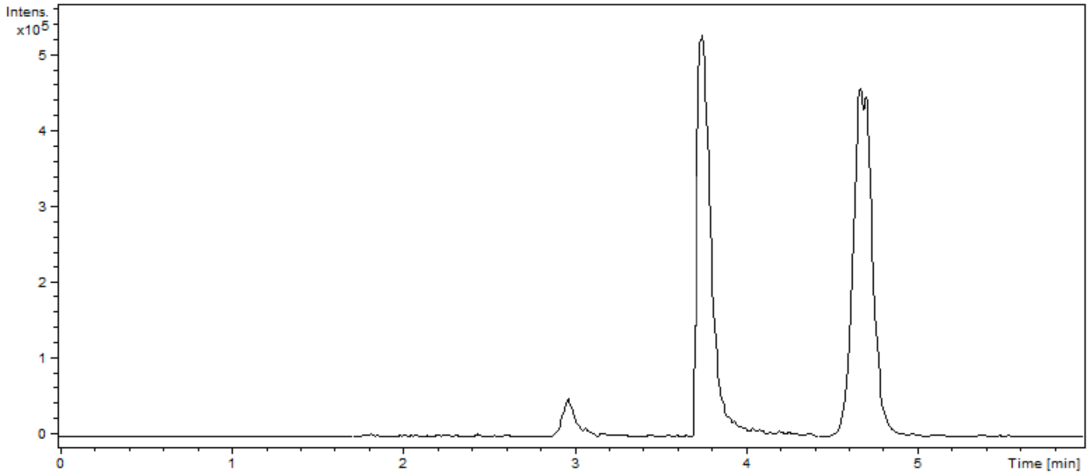

Figure S5 (C): TIC for tocopherols (δ-tocopherol – R_T=3.3 min; γ-tocopherol – R_T=4.1 min; α-tocopherol – R_T=5.1 min)

Sample 6 – *E. palustre* aerial parts (EtOH 30%)

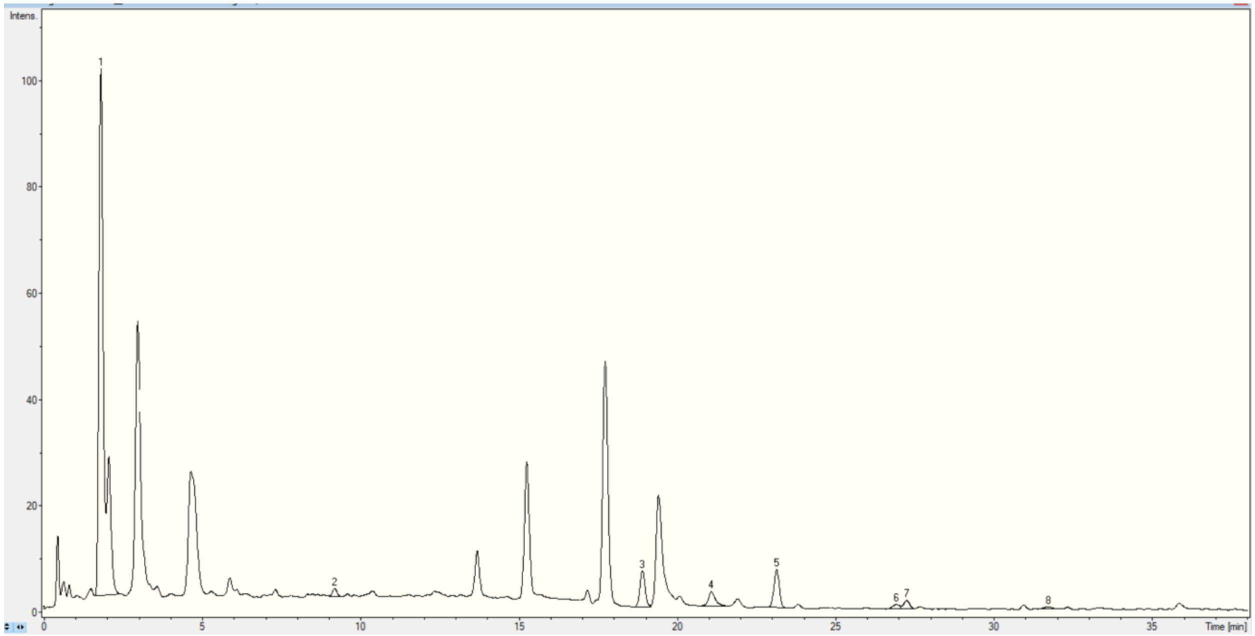

Figure S6 (A): The UV chromatogram for the individual polyphenolic compounds quantified by the first LC-MS method

Legend: 1- caftaric acid; 2- *p*-coumaric acid; 3- hyperoside; 4- myricetin; 5- quercitrin; 6- quercetol; 7- kaempferol-3-rhamnoside; 8- kaempferol.

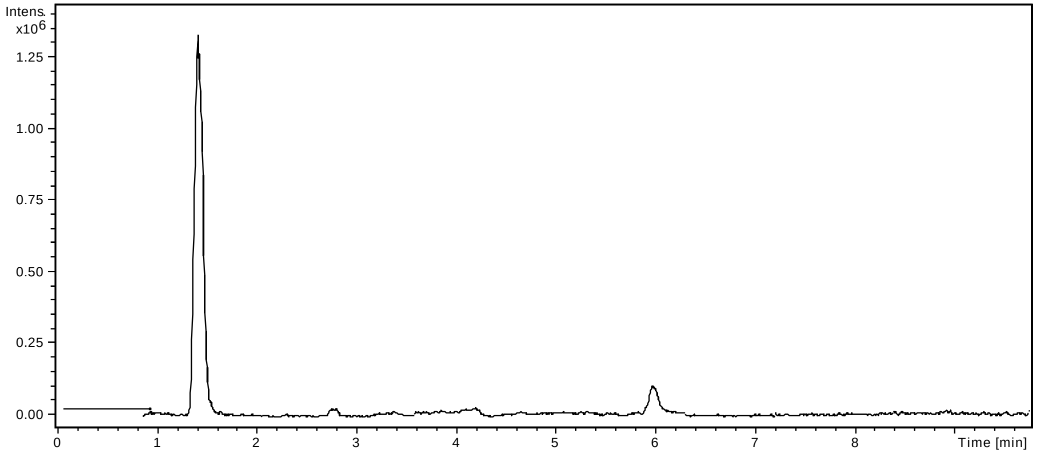

Figure S6 (B): The TIC for polyphenols analyzed with the second analytical method (Gallic acid – R_T=1.5 min; Protocatechuic acid – R_T=2.8 min; Catechin – R_T=6.0 min; Vanilic acid – R_T=6.7 min; Syringic acid – R_T=8.4 min; Epicatechin – R_T=9.0 min)

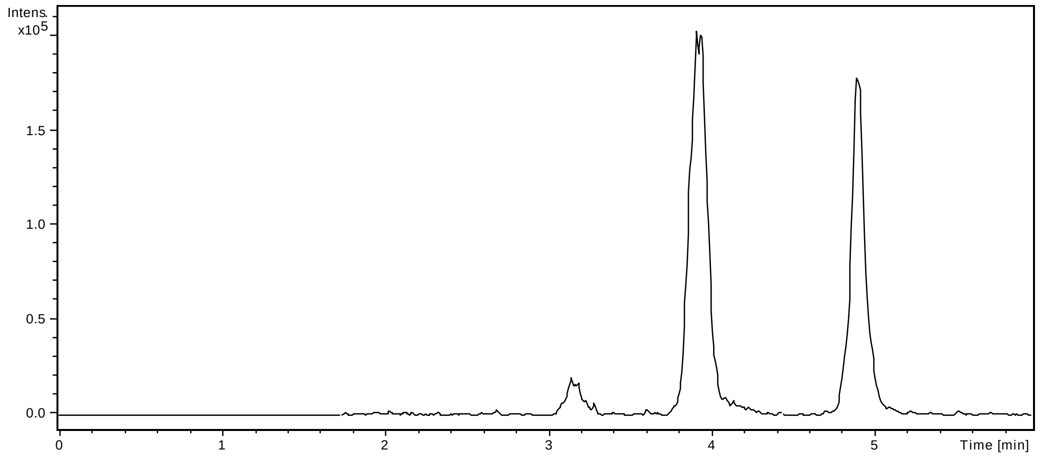

Figure S6 (C): TIC for tocopherols (δ-tocopherol – R_T=3.3 min; γ-tocopherol – R_T=4.1 min; α-tocopherol – R_T=5.1 min)

Sample 7 – *E. dodonaei* aerial parts (EtOH 30%)

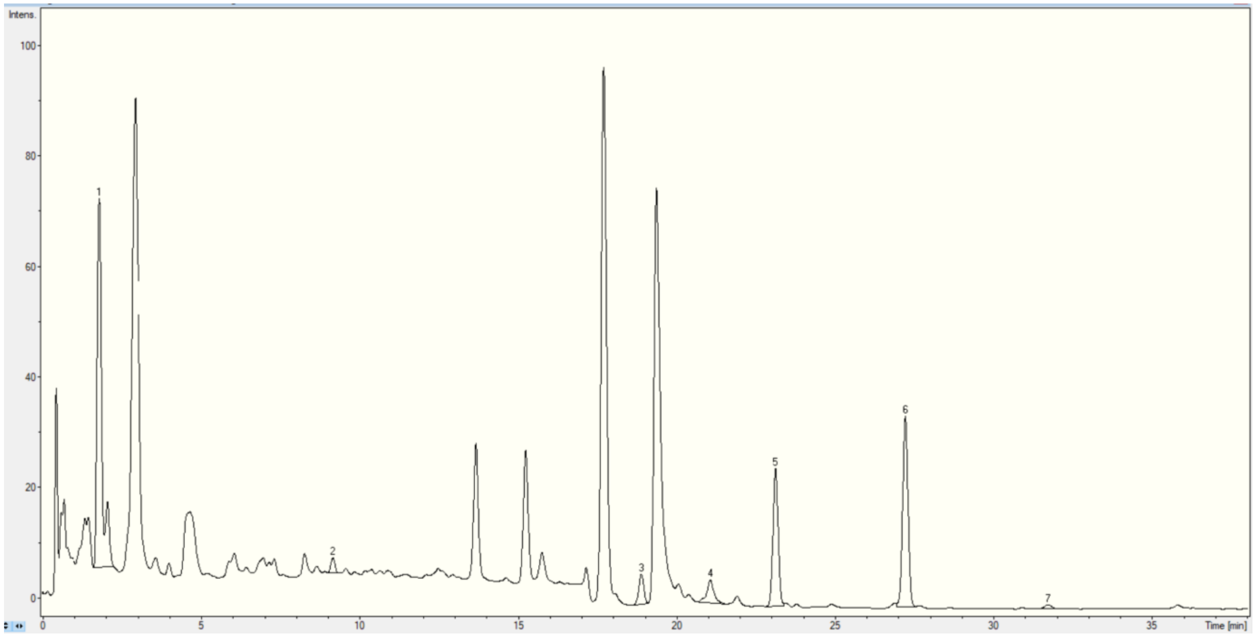

Figure S7 (A): The UV chromatogram for the individual polyphenolic compounds quantified by the first LC-MS method

Legend: 1- caftaric acid; 2- *p*-coumaric acid; 3- hyperoside; 4- myricetin; 5- quercitrin; 6- kaempferol-3-rhamnoside; 7- kaempferol.

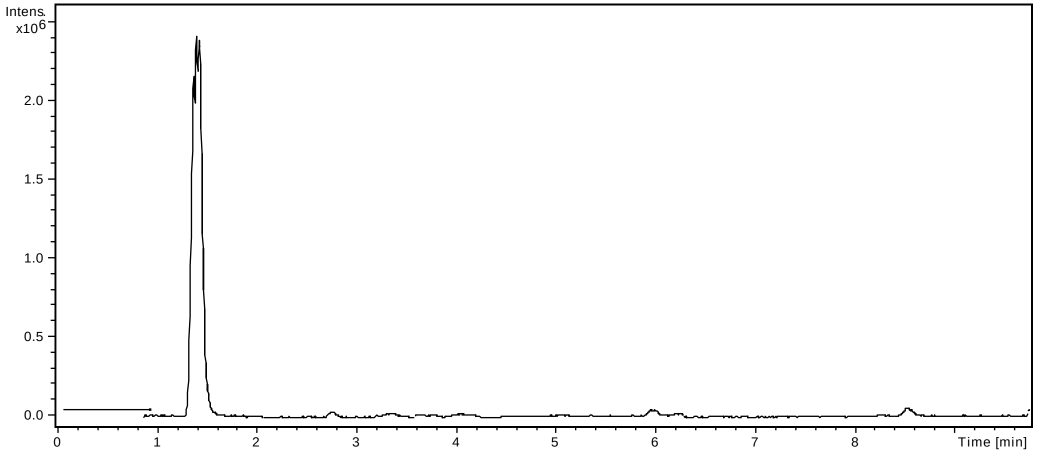

Figure S7 (B): The TIC for polyphenols analyzed with the second analytical method (Gallic acid – R_T=1.5 min; Protocatechuic acid – R_T=2.8 min; Catechin – R_T=6.0 min; Vanilic acid – R_T=6.7 min; Syringic acid – R_T=8.4 min; Epicatechin – R_T=9.0 min)

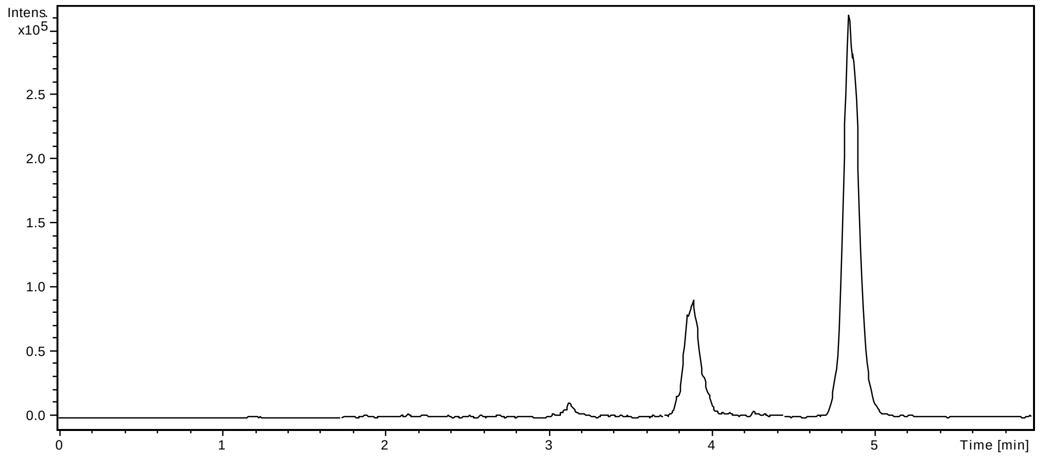

Figure S7 (C): TIC for tocopherols (δ-tocopherol – R_T=3.3 min; γ-tocopherol – R_T=4.1 min; α-tocopherol – R_T=5.1 min)

Sample 8 – *E. angustifolium* leaves (EtOH 30%)

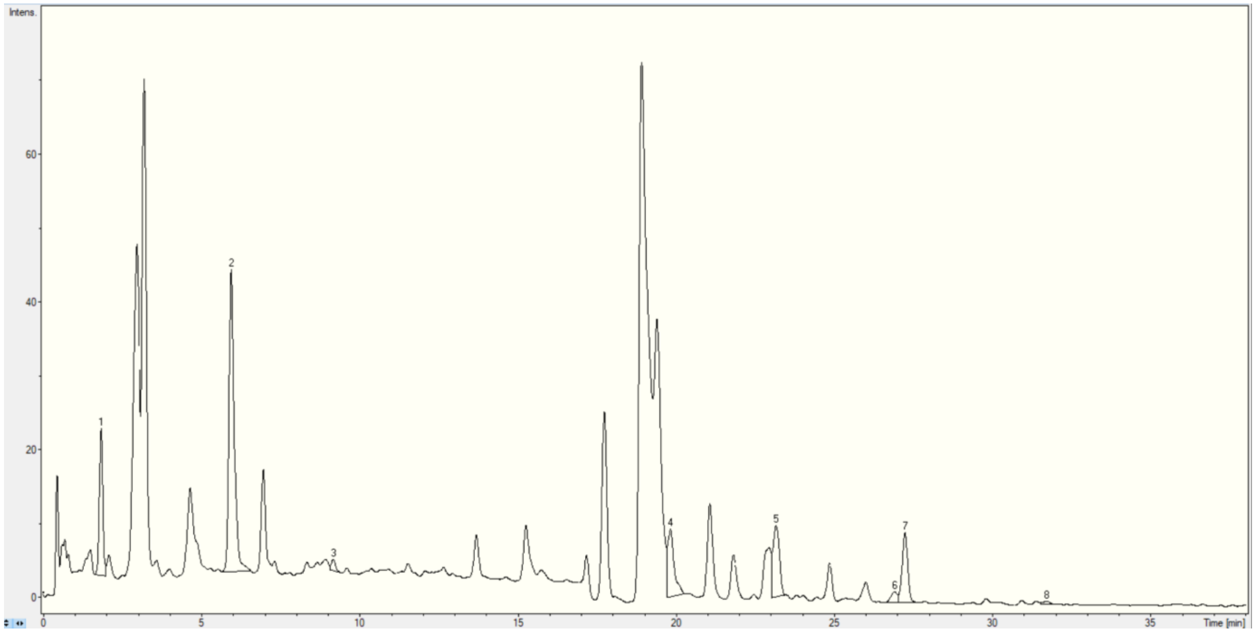

Figure S8 (A): The UV chromatogram for the individual polyphenolic compounds quantified by the first LC-MS method

Legend: 1- caftaric acid; 2- chlorogenic acid; 3- *p*-coumaric acid; 4- isoquercitrin; 5- quercitrin; 6- quercetol; 7- kaempferol-3-rhamnoside; 8- kaempferol.

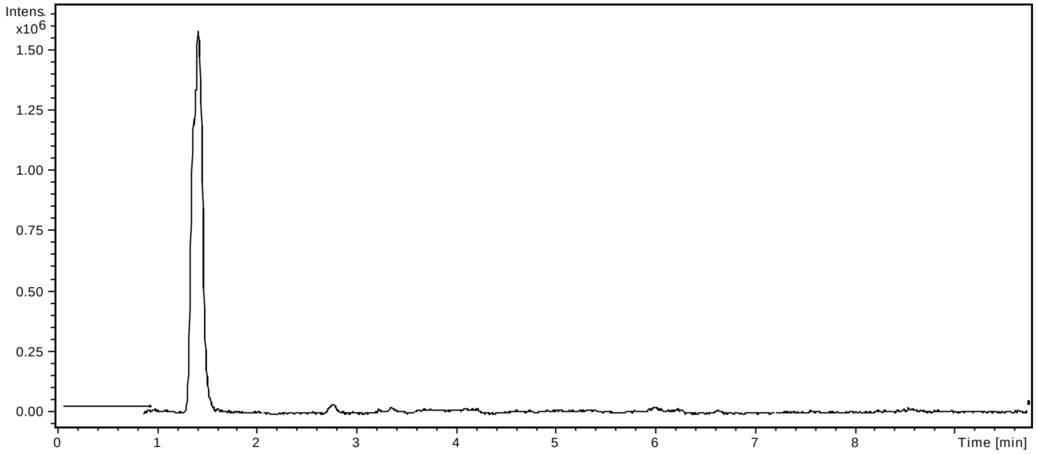

Figure S8 (B): The TIC for polyphenols analyzed with the second analytical method (Gallic acid – R_T=1.5 min; Protocatechuic acid – R_T=2.8 min; Catechin – R_T=6.0 min; Vanilic acid – R_T=6.7 min; Syringic acid – R_T=8.4 min; Epicatechin – R_T=9.0 min)

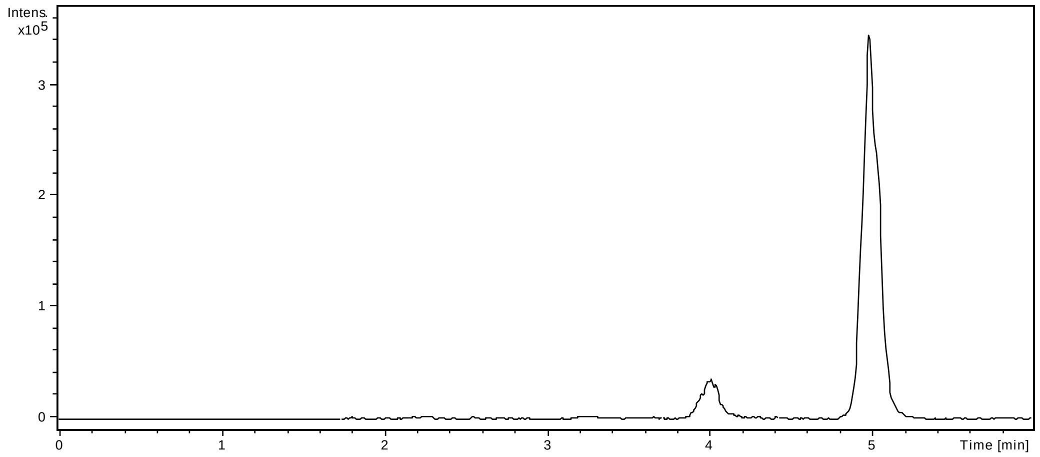

Figure S8 (C): TIC for tocopherols (δ-tocopherol – R_T=3.3 min; γ-tocopherol – R_T=4.1 min; α-tocopherol – R_T=5.1 min)

Sample 9 – *E. angustifolium* aerial parts (EtOH 30%)

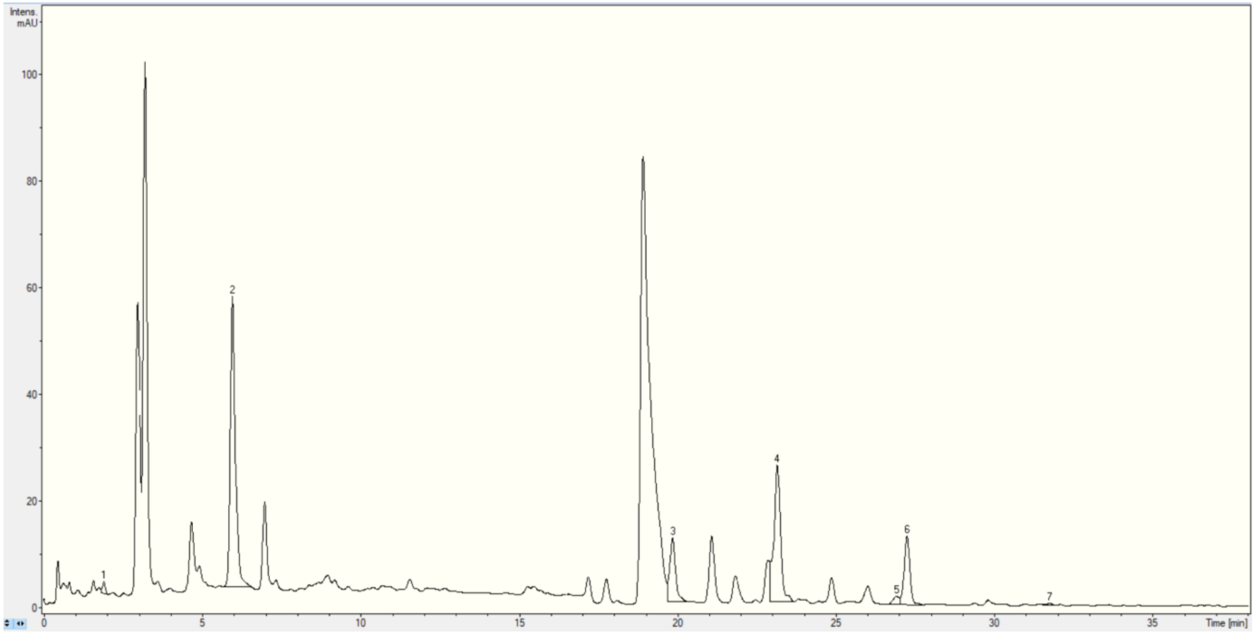

Figure S9 (A): The UV chromatogram for the individual polyphenolic compounds quantified by the first LC-MS method

Legend: 1- caftaric acid; 2- chlorogenic acid; 3- isoquercitrin; 4- quercitrin; 5- quercetol; 6- kaempferol-3-rhamnoside; 7- kaempferol.

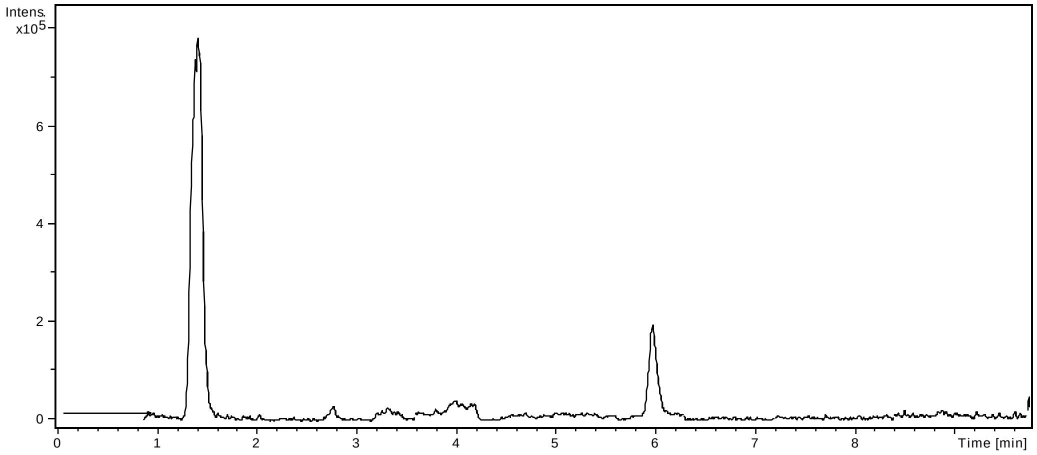

Figure S9 (B): The TIC for polyphenols analyzed with the second analytical method (Gallic acid – R_T=1.5 min; Protocatechuic acid – R_T=2.8 min; Catechin – R_T=6.0 min; Vanilic acid – R_T=6.7 min; Syringic acid – R_T=8.4 min; Epicatechin – R_T=9.0 min)

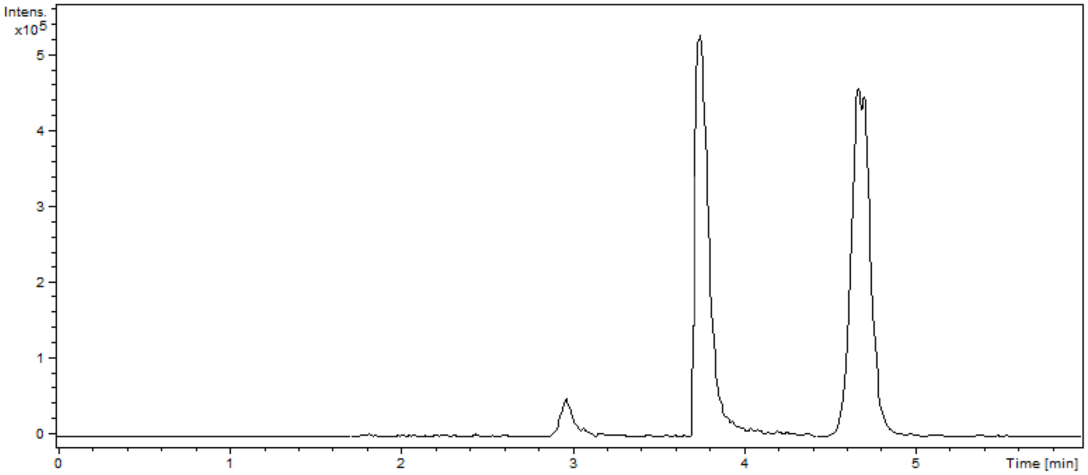

Figure S9 (C): TIC for tocopherols (δ-tocopherol – R_T=3.3 min; γ-tocopherol – R_T=4.1 min; α-tocopherol – R_T=5.1 min)
